# Upregulation of carbonic anhydrase 1 beneficial for depressive disorder

**DOI:** 10.1186/s40478-023-01545-6

**Published:** 2023-04-03

**Authors:** Ke Cheng, Yue Wang, Yong He, Yu Tian, Junjie Li, Chong Chen, Xingzhe Xu, Zhonghao Wu, Heming Yu, Xiangyu Chen, Yili Wu, Weihong Song, Zhifang Dong, Huatai Xu, Peng Xie

**Affiliations:** 1 NHC Key Laboratory of Diagnosis and Treatment on Brain Functional Diseases, The First Affiliated Hospital of Chongqing Medical University, Chongqing, 400016 China; 2 Institute of Neuroscience, State Key Laboratory of Neuroscience, CAS Center for Excellence in Brain Science and Intelligence Technology, Chinese Academy of Sciences, Shanghai, 200031 China; 3 Department of Neurology, The First Affiliated Hospital of Chongqing Medical University, Chongqing, 400016 China; 4 Ministry of Education Key Laboratory of Child Development and Disorders, National Clinical Research Center for Child Health and Disorders, Chongqing, 400014 China; 5 Chongqing Key Laboratory of Translational Medical Research in Cognitive Development and Learning and Memory Disorders, Children’s Hospital of Chongqing Medical University, Chongqing, 400014 China; 6 Oujiang Laboratory (Zhejiang Lab for Regenerative Medicine, Vision and Brain Health), Institute of Aging, Zhejiang Provincial Clinical Research Center for Mental Disorders, Key Laboratory of Alzheimer’s Disease of Zhejiang Province, School of Mental Health and Kangning Hospital, Wenzhou Medical University, Wenzhou, Zhejiang 325000 China; 7 Shanghai Center for Brain Science and Brain-Inspired Intelligence Technology, Shanghai, 201210 China

**Keywords:** Carbonic anhydrase 1, Hippocampal astrocytes, Miniature inhibitory postsynaptic currents, CAR1-knockout mice, Major depressive disorder

## Abstract

Carbonic Anhydrase 1 (CAR1) is a zinc-metalloenzyme that catalyzes the hydration of carbon dioxide, and the alteration of CAR1 has been implicated in neuropsychiatric disorders. However, the mechanism underlying the role of CAR1 in major depressive disorder (MDD) remains largely unknown. In this study, we report the decreased level of CAR1 in MDD patients and depression-like model rodents. We found that CAR1 is expressed in hippocampal astrocytes and CAR1 regulates extracellular bicarbonate concentration and pH value in the partial hilus. Ablation of the *CAR1* gene increased the activity of granule cells via decreasing their miniature inhibitory postsynaptic currents (mIPSC), and caused depression-like behaviors in *CAR1*-knockout mice. Astrocytic CAR1 expression rescued the deficits in mIPSCs of granule cells and reduced depression-like behaviors in CAR1 deficient mice. Furthermore, pharmacological activation of CAR1 and overexpression of CAR1 in the ventral hippocampus of mice improved depressive behaviors. These findings uncover a critical role of CAR1 in the MDD pathogenesis and its therapeutic potential.

**Supplementary Information:**

The online version contains supplementary material available at 10.1186/s40478-023-01545-6.

## Introduction

The mechanism underlying the pathogenesis of major depressive disorder (MDD) remains elusive. Many factors, including genetic abnormalities, monoamine deficiency, HPA axis hyperactivity, defective neurogenesis, GABA disturbances, neuro-inflammation, and microbiome perturbation are associated with MDD [18, 39]. Dysfunction of neural circuits in the hippocampal dentate gyrus (DG) was reported to cause depression-like behaviors [23, 29, 38]. Recent studies further showed that the hyperexcitability of granule cells (GCs) in DG results in depression-like behaviors [1, 20]. However, the mechanism underlying dysfunctional DG neural circuit in MDD remains largely unknown.

Altered Carbonic Anhydrase 1 (CAR1) in the frontal cortex was detected in the MDD postmortem tissue [15]. CAR1 is a zinc-metalloenzyme that catalyzes the reversible hydration of CO_2_ [24]. Activation of carbonic anhydrase (CAR) converts carbon dioxide and water into bicarbonate ions (HCO3^−^) and protons (H^+^) [19]. Decreased CAR reduces the concentration of extracellular bicarbonate ions and protons, resulting in an alkalized extracellular environment. CAR family orchestrates the balance of neural excitability and synaptic transmission via regulating the release of bicarbonate and proton (H^+^) into the extracellular space [7]. H^+^ could directly affect synaptic transmission [8, 9], and abnormal acidity in the amygdala was demonstrated to trigger intense fear, anxiety and depression-like behaviors [6, 35, 40]. CAR disruption results in impairment of stress-experienced memories that might lead to MDD [30]. Postmortem studies also suggested that the pH disturbance is implicated in psychiatric disorders [13, 14]. CAR4 and CAR14 are the main isoforms in the brain and mediate Cl^−^–HCO_3_^−^ exchange in hippocampal neurons [34]. CAR2 and CAR7 could cause febrile seizures via pH mediation [28]. However, the role of CAR1 in MDD has not being well examined. We found that CAR1 is expressed in the hippocampus, and CAR1 but not other CAR family members modulates bicarbonate concentration and pH in the part of hilus to mediate the excitability of GCs by affecting their inhibitory neuronal transmission; and CAR1 deficiency leads to depression-like behavior. Furthermore, restoration of CAR1 expression in astrocytes of CAR1 deficient mice rescued the deficits in mIPSCs of granule cells and reduced depression-like behaviors. Finally, pharmacological activation and overexpression of CAR1 in the ventral hippocampus of mice alleviates depressive behaviors. These findings uncover a critical role of CAR1 in the pathogenesis of MDD and its therapeutical potential for treating depressive disorders.

## Results

### Decreased expression of CAR1 in MDD patients and depression-like model rodents

**Fig. 1:**
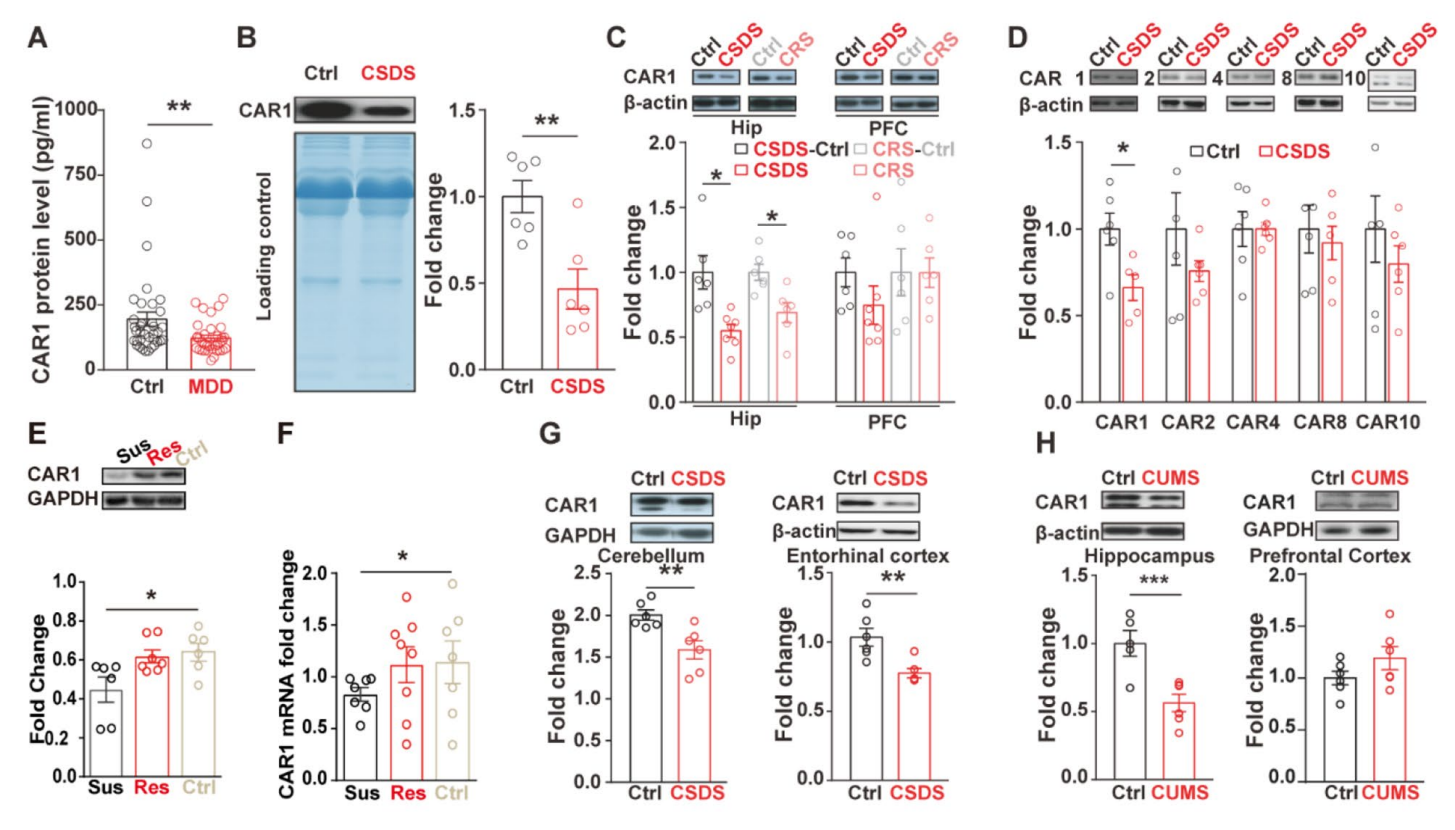
Decreased expression of CAR1 in MDD patients and depression-like model rodents. **(A)** The protein level of CAR1 in healthy controls (n = 29) and MDD patients (n = 36) revealed by ELISA assay. **(B)** Western blot analysis for the expression level of CAR1 in the serum of mice after CSDS exposure. **(C)** Western blot analysis showed the changes of CAR1 in the hippocampus and prefrontal cortex of mice after CSDS or CRS (n = 6 for each group) treatment. **(D)** Western blot analysis of CA family isoforms in the hippocampi between CSDS mice model and control mice group. **(E)** Western blot analysis showed the changes of CAR1 in the hippocampus among CSDS susceptible group (n = 6), resilient group (n = 7) and control group (n = 6). **(F)** Q-PCR identification of car1 mRNA in the hippocampi among among CSDS susceptible group (n = 7), resilient group (n = 8) and control group (n = 7). **(G)** Western blot analysis showed the changes in the cerebellum and entorhinal cortex of CSDS mice. **(H)** The expression of CAR1 in the hippocampus and prefrontal cortex from the CUMS-treated rats. β-actin or GAPDH was used as loading controls. Unpaired *t*-test analysis, *p < 0.05, **p < 0.01

To investigate the association of CAR1 with depressive disorders, we first measured the plasma concentration of CAR1 in MDD patients and found that CAR1 level was significantly lower in the patients (n = 36) comparing to healthy controls(n = 29) (Fig. 1A). To explore the potential role of CAR1 in the pathogenesis of depression, the depression-like mouse models with chronic social defeat stress (CSDS) [36] and chronic restraint stress (CRS) were developed. After 10 days the CSDS model mice displayed longer immobile time in the Forced Swimming Test (FST) and decreased social interaction (Fig. S1A), while CRS model mice showed decreased sucrose consumption and increased immobile time in FST comparing to control groups (Fig. S1B). The serum CAR1 level was decreased in the CSDS mice comparing to the control group (Fig. 1B) but not in CRS mice (Fig. S1C). The expression level of CAR1 in different regions of the brain, including the hippocampus and the prefrontal cortex (PFC) was further examined by Western blotting. CAR1 was significantly lower in the hippocampus, but not in the PFC after CSDS or CRS model mice (Fig. 1C), while other brain enriched isoforms of CAR2, 4, 8 and 10 remained unchanged (Fig. 1D). Furthermore, the CAR1 protein expression was significantly decreased comparing to control group, while there is unchanged CAR1 expression in the resilient group. As a parallel, the mRNA level of CAR1 was also significantly decreased in the susceptible group but not in the resilient group comparing to the control group (Fig. 1E, F). CAR1 was also lower in the cerebellum and the entorhinal cortex (Fig. 1G) but had no significant change in the choroid plexus, hypothalamus, cecum, or neocortex of CSDS mice (Fig. S1D), neither of non-significant change between female CRS mice comparing to control (Fig. S1E), indicating that chronic stress induced a brain regional-specific changes in CAR1. By using depression model rats exposed to chronic unpredictable mild stress [5], we also confirmed the reduction of CAR1 in the hippocampus, but not in the prefrontal cortex (Fig. 1H). The data suggested that lower CAR1 level in the brain is associated with depression-like behaviors.

### Cell-specific expression of CAR1 in hippocampal astrocytes

**Fig. 2:**
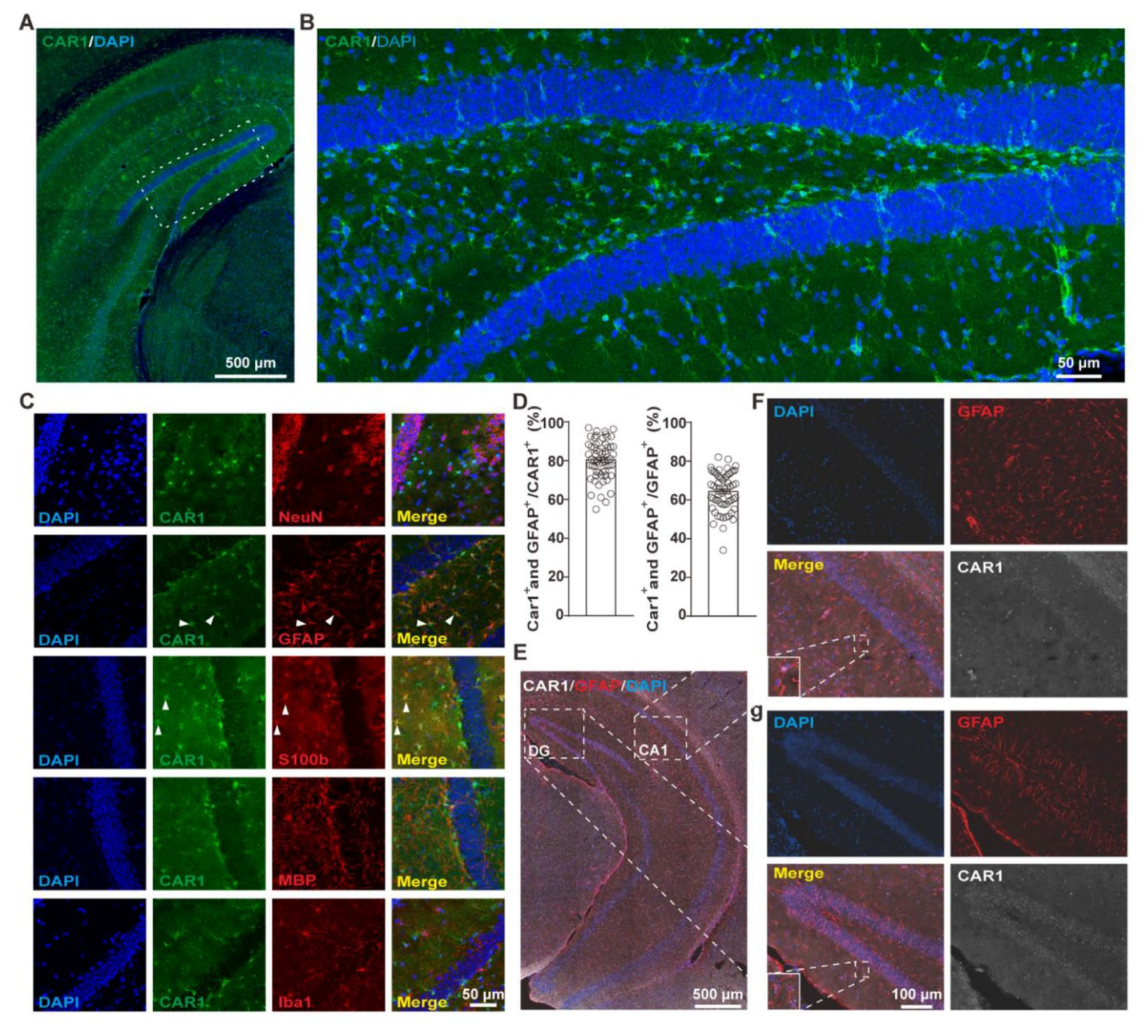
Specific expression of CAR1 in astrocytes. **(A, B)** A sample confocal image of a hippocampal slice stained with antibody against CAR1 (green) and DAPI (blue). Boxed area in A was zoomed in B. **(C)** Co-immunostaining with antibodies against CAR1 (green) and different cell type markers (red). Solid arrowheads: representative double-labeled cells. **(D)** The quantification of CAR1^+^ /GFAP^+^ cells in CAR1^+^ cells or GFAP^+^ cells. **(E)** RNAscope signals of CAR1 (white) in astrocytes labeled by anti-GFAP immunostaining (red) of the ventral hippocampus. **(F, G)** Zoomed in figures from E. F for the CA1 region and G for the DG region

The spatial expression of CAR1 in the brain has not been previously well examined. Hippocampus is a region implicated in MDD pathogenesis [21]. CAR1 expression was detected in the hippocampus (Fig. 2A and B). Immunostaining assay with cell-type-specific markers (GFAP, NeuN, S100β, Iba1, and MBP) showed that CAR1 was specifically co-labeled with markers of astrocytes, but not of neurons, oligodendrocytes, or microglial cells in the dentate gyrus (Fig. 2C). CAR1 expression was mainly detected in astrocytes of CA1 (Fig. 2A and Fig. S2). ~80% of CAR1^+^ cells were expressing GFAP and CAR1 was expressed in ~ 70% GFAP^+^ cells in the hippocampus (Fig. 2D). The RNAscope assay confirmed the expression of CAR1 at the transcription level in astrocytes (Fig. 2E-G). These data demonstrate that CAR1 is expressed in hippocampal astrocytes.

### Ablation of CAR1 caused depression-like behaviors in the CAR1 deficient mice

**Fig. 3:**
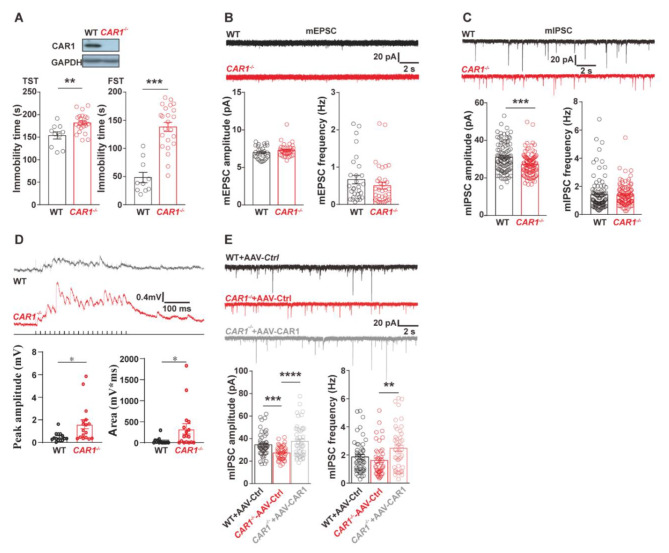
A causal role of CAR1 in depression-like behaviors and the activity changes in granule cells. **(A)** Western blotting of hippocampal sample to validate the absence of CAR1 in *CAR1*^***−/−***^ mice(top); depression-like behavioral tests including FST and TST for *CAR1*^***−/−***^ mice and WT mice (bottom). **(B, C)** Example traces and their average amplitude and frequency of mEPSC **(D)** or mIPSC **(E)** in DG granule cells. **(D)** Representative responses of granule cells (black, WT; red, *CAR1*^***−/−***^) to perforant pathway stimulations (top); the peak response and the area under the curve quantification (bottom) of evoked responses (WT, n = 14; *CAR1*^***−/−***^, n = 17). **(E)** The representative mIPSC traces of DG granule cells from WT mice injected with AAV-control, CAR1^**−/−**^ mice with AAV-control, or AAV-CAR1 (left), and their average amplitude and frequency of mIPSC of DG granule cells (right). *p < 0.05, **p < 0.01, ***p < 0.001, ****p < 0.0001 (Student’s *t-test* and one-way ANOVA)

**Fig. 4:**
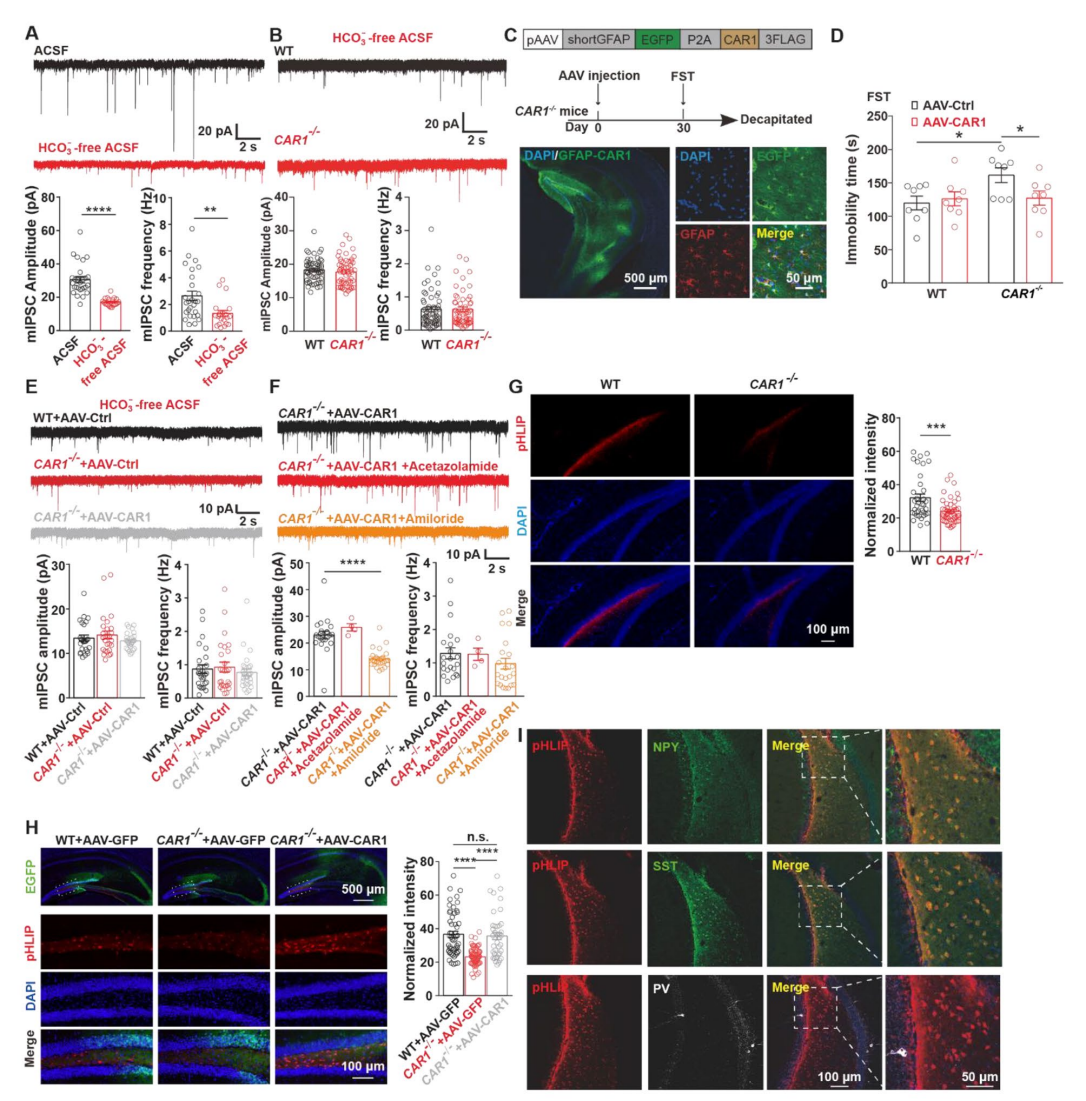
Decreased the concentration of bicarbonate and proton in the extracellular space of the hilus of *CAR1*^***−/−***^ mice. **(A)** Example mIPSC traces (top) and their average amplitude and frequency (bottom) of DG granule cells from WT mice underHCO_3_^−^-free ACS for the normal ACSF condition. **(B)** Example mIPSC traces (top) and their average amplitude and frequency (bottom) of DG granule cells from WT mice and *CAR1*^*−/−*^ mice under HCO_3_^−^-free ACSF condition. **(C)** Schematic representation of a construct (top) and the timeline of the experimental procedure (middle). A representative image of the vHPC after bilateral infusions of AAV (bottom left). The infection of astrocytes was confirmed by co-immunostaining with EGFP (green) and GFAP (red) (bottom right). **(D)** The immobility time in the FST test after AAV-CAR1 infection. *p < 0.05, **p < 0.01 (Student’s *t-test*). **(E)** Representative mIPSC traces of DG granule cells from WT mice injected with AAV-control, CAR1^**−/−**^ mice with AAV-control or AAV-CAR1 underHCO_3_^−^-free ACSF condition (top) and their average amplitude and frequency analysis (bottom). **(F)** mIPSC traces of DG granule cells after CAR1 overexpression in*CAR1*^*−/−*^astrocytes with control, acetazolamide, and amiloride treatment as well as their average amplitude and frequency quantifications (bottom). **(G)** Representative images in the DG hilus after bilateral infusions of pHLIP at the vHPC (n = 4) (left); fluorescence density quantification of pHLIP labeled hilus interneurons from WT and *CAR1*^*−/−*^ mice (right). **(H)** Representative images in the DG hilus after bilateral infusions of pHLIP at vHPC into WT mice infected with AAV-GFP, *CAR1*^*−/−*^ mice with AAV-GFP or AAV-CAR1 and their fluorescence density quantification; boxed regions in the top panels were zoomed in the bottom panels. **(I)** The validation of pHLIP marked hilus interneuron identities by co-immunostaining with neuropeptide Y (NPY), Somatostatin (SST), and parvalbumin (PV). *p < 0.05, **p < 0.01, ***p < 0.001 (Student’s *t-test* and one-way ANOVA)

**Fig. 5:**
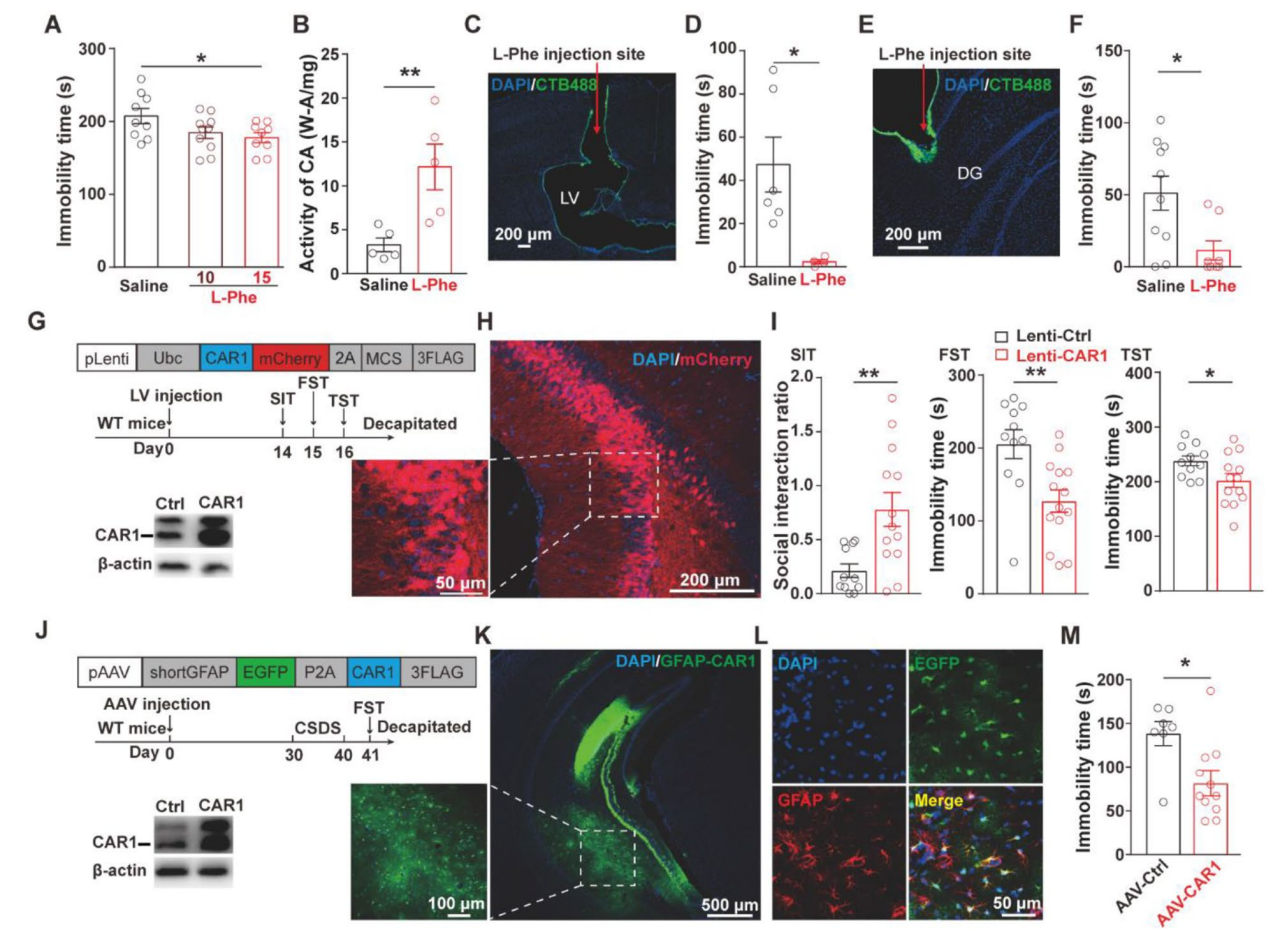
Pharmacological activation of CAR or overexpression of CAR1 in vHPC astrocytes promoted anti-depression-like behavior. **(A)** The immobility time of FST after intraperitoneal injection with saline (n = 9) or L-Phe (10 mg/kg, n = 10; 15 mg/kg, n = 9). **(B)** Enzyme activity of CAR in the hippocampus after injecting saline (n = 5) and L-Phe (15 mg/kg, n = 5). **(C, E)** The images to show the cannula traces into the lateral ventricle (C) or into the vHPC (E). **(D, F)** The immobility time in FST 30 min after injection into the lateral ventricle (L-Phe2.5ug/ul, n = 4; saline, n = 6, D) or into the vHPC(L-Phe25 µg/side, n = 10; saline, n = 9, F). **(G)** Schematic representation of a construct andthe timeline of the experimental procedure; the bottom panel shows the overexpression with the Western blotting. **(H)** A representative confocal image after lenti-viral injection in the vHPC. **(I)** Behavior tests after CAR1 overexpression. **(J)** Schematic representation of an AAV construct with GFAP promoter and the timeline of experimental procedure; the bottom panel shows the overexpression with the Western blotting. **(K)** A representative confocal image of the vHPC after AAV injection. **(L)** The infection of astrocytes was confirmed by co-immunostaining with EGFP (green) and GFAP (red). **(M)** The immobility time in FST (GFAP-CAR1, n = 11; GFAP-Control, n = 7) after CSDS treatment

To determine the causal role of CAR1 in the pathogenesis of depression, we generated the CAR1-knockout (*CAR1*^*−/−*^) mice (Fig. S3). Ablation of CAR1 increased the immobile time in FST and TST in *CAR1*^*−/−*^ mice compared with WT mice (Fig. 3A), suggesting the absence of CAR1 leads to depression-like behaviors. Meanwhile, CAR1^−/−^ mice displayed decreased the time and distances in the open armed of EPM (Fig S4A, B), while increased the time spent in the EPM closed arms comparing to the WT mice (Fig. 4D), which indicates that CAR1 deficits at ventral hippocampus cause anxiety phenotypes. Also, there was no difference of total moving distances in OFT (Fig. 5E) either of anhedonia deficit via sucrose preference test between two groups (Fig. 5F). Western blot analysis of the tissue lysates from WT and *CAR1*^*−/−*^ mice also confirmed the specificity of the antibody against the CAR1 protein (Fig. 3A). Since CAR induced endogenous proton (H^+^) changes that could affect NMDA-mediated synaptic plasticity [12], whole-cell patch-clamp recordings were performed on acute hippocampal slices to examine whether the absence of CAR1 affects GCs on synaptic transmissions in the vDG. There were no significant changes in either amplitude or frequency of miniature EPSC (mEPSC) in *CAR1*^*−/−*^ mice compared with WT mice (Fig. 3B). Dysregulation of NMDAR2A (NR2A)- and NMDAR2B (NR2B)-mediated EPSC is implicated in the development of MDD [37]. *CAR1*^*−*/−^ mice exhibited no significant change in the current ratio of AMPAR/NMDAR or NR2A/NR2B (Fig. S6), indicating that CAR1 deficiency did not affect glutamatergic synaptic transmission. However, the amplitude of mIPSC of GC was significantly decreased in *CAR1*^*−/−*^mice compared with control mice (Fig. 3C). Consistent with this effect, recordings from GCs showed increased evoked responses to the perforant pathway stimulation in *CAR1*^*−/−*^mice, indicating the excitability of GCs was significantly increased (Fig. 3D). Meanwhile, neither mEPSC nor mIPSC of pyramidal cells in CA1 was significantly altered in the *CAR1*^*−/*−^ mice (Fig. S7). Remarkably, restoration of CAR1 expression in astrocytes increased both the amplitude and frequency of GCs’ mIPSC (Fig. 3E). We also detected an enhanced long-term potentiation of the Schaffer collateral pathway (CA3-CA1) downstream of the DG in *CAR1*^*−/−*^ mice compared with WT mice (Fig. S8). These data suggest that the ablation of CAR1 induced depressive behavior, and decreased inhibition and increased activity of GCs in the DG of *CAR1*^*−/−*^ mice.

### Disruption of CAR1 decreased hippocampal bicarbonate and proton levels

To examine the mechanism underlying the dependence of bicarbonate ions for the decreased mIPSC in *CAR1*^*−/*−^ mice. CO_2_/HCO_3_^−^-buffer was replaced with bicarbonate-free solution (HEPES buffer saturated with O_2,_ without HCO_3_^−^). Switching to HEPES buffer significantly reduced both frequencies and amplitudes of mIPSC in GCs (Fig. 4A), abolished the differences in mIPSC of GCs between *CAR1*^*−/−*^ and control groups (Fig. 4B). We have shown that CAR1 is expressed in hippocampal astrocytes (Fig. 2); Astrocytic CAR1 played an important role in causing depression-like behaviors. As shown in (Fig. 4), expression of CAR1 in astrocytes increased both the amplitude and frequency of GCs’ mIPSC. To further examine CAR1’s beneficial effect on depression-like behaviors, CAR1 was specifically expressed in astrocytes in the vDG of *CAR1*^*−/−*^ mice (Fig. 4C). Four weeks after viral expression in mice, re-expression of CAR1 in astrocytes reduced the immobility time in FST compared with the control group in CAR1-/- mice (AAV-Ctrl, n = 8; AAV-CAR1, n = 8; p < 0.05) but not in the WT mice (AAV-Ctrl, n = 8; AAV-CAR1, n = 8; Fig. 4C, D). Under this circumstances, blocked the rescue effect of the restoring astrocytic expression of CAR1 in the *CAR1*^*−/−*^ mice (Fig. 4E). This observation suggested that reducing concentration of extracellular bicarbonate ions by disruption of CAR1 decreased mIPSC in the DG of the mice. To further confirm the effect, acetazolamide (20µM), a potent inhibitor for CAR2, 4, 5, 7, 9 but not CAR1 [31], was applied to block HCO_3_^−^ regeneration. Acetazolamide treatment did not affect the mIPSC of GCs, indicating that the decreased mIPSC amplitude in GCs by CAR1 ablation was CAR1-dependent (Fig. 4F). Amiloride is a potent blocker of neuronal Na^+^-H^+^ exchanger that could alkalinize the extracellular environment [32]. To address the potential role of protons, amiloride was applied during the recording. Application of amiloride significantly blocked the CAR1’s rescuing effect on the amplitude of mIPSC of GCs in *CAR1*^*−/−*^ mice. The data suggest that CAR1 deficiency resulted in the alkalized extracellular condition in the DG of *CAR1*^*−/−*^ mice (Fig. 4G).

Next, a fluorescent dye pHLIP was used to directly examine CAR1’s effect on the potential alteration of pH in the DG. pHLIP is a transmembrane helix pH-Low Insertion Peptide with a fluorophore and a more acidic environment makes pHLIP easier to enter the cytoplasm in cells [2, 27] (Fig. S9). We found less fluorescent signals inside cells in the hippocampal hilar region of *CAR1*^*−/−*^ mice (WT, 36 slices from 4 mice; *CAR1*^*−/−*^, 46 slices from 4 mice; p < 0.01) (Fig. 4E). Restoring expression of CAR1 in astrocytes in *CAR1*^*−/−*^ mice increased the fluorescence level in hilar cells (WT AAV-GFP 54 slices from 3 mice; *CAR1*^*−/−*^ AAV-GFP 68 slices from 3 mice; *CAR1*^*−/−*^ AAV-GFP 40 slices from 3 mice) (Fig. 4G). We further confirmed that those fluorophore-positive neurons in the hippocampal hilus were SST/NPY interneurons (Fig. 4H, I). These results strongly suggested that the absence of CAR1 could alkalize the local milieu at the hippocampal hilus region. Finally, we aimed to explore the pH manipulation at vDG whether related to depressive like behaviors, there is no behavioral differences between CAR1^−/−^ mice (CAR1^−/−^ -pH7.4 vs. CAR1^−/−^-Ph 6.0) and WT mice (WT-pH7.4 vs. WT-pH 6.0) after cannula infusion of acid for 7 days (Fig. S10). This data could be parsimoniously interpreted that the CAR1 deletion brings in limited and local changes in extracellular pH, while the paradigm in the context was dramatically changed, which inevitably triggered acid sensitive ion channels and receptors, which may be partially accounted for the negative behavior data hereby. Also, the optimal volume and behavior tests timing requires to further explore in the future.

### Upregulation of CAR1 in hippocampal astrocytes inhibited depression-like behaviors

To assess the effect of CAR1 on depression, CSDS model mice were intraperitoneally injected with a CAR agonist, L-phenylalanine (L-Phe) [3, 22] at 15 mg/kg once daily for five weeks. L-Phe treatment significantly decreased the immobile time in FST (Fig. 5A), and increased the CAR activity in the hippocampus (Fig. 5B). Next, L-Phe was bilaterally injected into the lateral ventricle (LV) (2.5 µg/side) or the ventral dentate gyrus (vDG) (25 µg/side) for 10 days (Fig. 5C and E). Both treatments significantly decreased the immobile time of the mice comparing to the saline group (Fig. 5D and F). These results suggest that the activation of CAR had an anti-depressant effect. To further investigate the potential effect of CAR1 on depression-like behaviors, lenti-viral vectors overexpressing *CAR1* was microinjected into the ventral hippocampus [10]. Overexpression of CAR1 in the vDG (Fig. 5G and H) increased the social interaction time (Ctrl, n = 11; CAR1, n = 13; p < 0.01) and decreased the immobile time in both TST (Ctrl, n = 11; CAR1, n = 14; p < 0.05) and FST (Ctrl, n = 11; CAR1, n = 13; p < 0.01) (Fig. 5I). Furthermore, mice were infected with GFAP-AAVs to express CAR1 in astrocytes in vDG and then subjected to CSDS (Fig. 5J-M), while CAR1 knockdown via GFAP-AAVs, showed no significant differences in depressive like behaviors, but spent less time in center (Open field test) comparing to GFAP-control group (Fig. S11), this might be related to anxiety like phenotypesOverexpression of CAR1 but not CAR2 (Fig. S12) significantly reduced immobile time in FST comparing to the control group (Fig. 5M). However, overexpression of CAR1 with neuron-targeted SYN-AAV failed to improve depression-like behaviors (Fig. S13). Overexpression of CAR1 with AAV5 serotype vector in the choroid plexus (CP) [4] did not affect depression-like behaviors with or without CSDS protocol treatment either (Fig. S14). These results demonstrated that upregulation of CAR1 by pharmacological activation or astrocyte-specific overexpression inhibits depressive-like behaviors.

These result further support that upregulation of astrocytic CAR1 could reverse the deficits of inhibitory synaptic transmission in the DG and improve the depression-like behavior in *CAR1*^*−/−*^ mice.

## Discussions

Accumulating evidences implicate DG in affective processing [17]. Activities of DG granule cell at the ventral hippocampus are tightly associated with depressive like behaviors. Inhibition of granule cells in the vDG confers resilience while excitation promotes susceptibility [1, 20]. In this study, we found that down regulation of CAR1 in the hippocampus caused the alkalization of the hilus region in the vDG, leading to the reduction of mIPSCs amplitude of granule cells and the induction of depression-like behaviors. We found that the CAR proteins are essential for maintaining the PH homeostasis in the brain. The fluctuation of pH value could affect neuronal membrane excitability, synaptic transmission, and signal cascades [25], this is in a line with prior report, inhibition of CAR isoforms could diminish the PH buffering capacity, thus affecting intracellular and extracellular PH and γ-aminobutyric acid (GABA) receptor function [11]. Moreover, local pH disorder in amygdala is thought to trigger the intense fear [6], anxiety [35]. Therefore, it is conceivable that the abnormal pH regulation is one of main reasons underlying the pathogenesis of neurological diseases. It is noteworthy that explicitly and directly manipulating pH in the vDG as well as other multiple brain region induces depressive like behaviors is intriguing. Inevitablly, we cannot exclude the possibility that acidosis activates some other pH-sensitive receptors or ion chanels, which requires future exploration.

Previous postmortem studies also indicated that the pH disturbance was implicated in psychiatric disorders [13, 14]. Consistent with the previous report that acidified condition could enhance the GABA signaling [8], our data showed that the disruption of astrocytic CAR1 could alkalize the hippocampal hilus region and impair the GABAergic transmission. Findings herein also indicate that CAR1 in astrocytes is a vital mediator regulating the activity of granule cells via affecting extracellular bicarbonate level and pH value in the hippocampus. CAR1 deficiency impairs the inhibitory neural transmission, therefore, increases granule cells activity in the DG, which leads to depression-like behaviors. Furthermore, we discovered upregulation of CAR1 in the astrocytes reduced depression-related phenotypes. Additionally, activation of CAR isoforms has been reported to be related to memory formation [33], spatial memory [16], the object recognition (OR) [3] test via regulating ERK phosphorylation in hippocampus. More compelling, the engagement of CAs is essential for providing the brain with the resilience necessary to ensure the consolidation of extinction under PTSD [30], which might involve in PTSD by ERK signaling pathway. Based on these evidences, it’s merit of exploiting the molecular mechanism of CAR1 involves in depressive like behaviors.

Finally, our results provide a novel insight into CAR1’s critical role in the pathogenesis of MDD and the beneficial effect of CAR1 upregulation on treating depression.

## Materials and methods

### Human subjects for ELISA assay

The project was approved by the ethics committee of Chongqing Medical University. The study enrolled thirty-five drug-naive patients with MDD (18 males and 17 females, average age = 26 ± 0.50) and thirty-six healthy controls (17 males and 19 females, average age = 25.63 ± 0.42) from the first affiliated hospital of Chongqing Medical University. All participants or their legally authorized representatives provided signed informed consent. The diagnose of MDD was according to the criteria described in the Diagnostic and Statistical Manual of Mental Disorders-version 4 (DSM-IV). Hamilton Depression Scale (17-Items) (HAMD-17) was used to quantify the severity of depression. We recruited first-episode drug-naive MDD patients (HAMD-17 scores ≥ 17). All participants did not have comorbidities of any known physical or other mental disorders. Information regarding sex, age, and depression symptoms severity of human subjects were collected (shown in table S2). The blood samples of fasting participants were collected with 5 ml vacutainer tubes prefilled with heparin lithium. Samples were centrifuged at 3000 rpm for 15 min (4 °C), and the supernatant was extracted. Plasma samples were stored at − 80 °C before the usage. ELISA of CAR1 were performed according to the manufacturer’s standard protocol (Cat: KL-CA1-Hu, Shanghai Kanglang).

### Animals

Animal experiments were approved by the Ethics Committee of the Chongqing Medical University or by the Animal Care and Use Committee of the Center for Excellence in Brain Science and Intelligence Technology/Institute of Neuroscience, Chinese Academy of Sciences. All treatments of animals follow the requirements of the National Institutes of Health Guidelines for Animal Research. All mice were group-housed under 12 h light-dark cycle (light on from 8 a.m. to 8 p.m.) with free access to water and food.

### *Car1*^−/−^ mice

We used the KO first strategy to get *Car1*^*−/−*^knockout mice (prepared by Nanjing Biomedical Research Institute of Nanjing University). BAC (bacterial artificial chromosome)-retrieval methods were used for constructing the *Car1* KO first targeting vector. In brief, we retrieved the *Car1* gene from a C57BL/6J BAC clone (provided by Sanger Institute) by a retrieval vector containing two homologous arms. After retrieving, this vector holds 11.0 kb of the genomic sequence, including 4.0 kb upstream of exon I, exon I-III, and part of intron III of the *Car1* gene. We inserted a cassette of FRT-En2-SA-IRES-LacZ-LoxP-Neo-FRT-loxP into intron II, 0.5 kb upstream of exon III, another single loxP into intron III, 0.3 kb downstream of exon III of the *Car1* gene. The targeting construct containing a neomycin expression cassette for positive selection and an HSV-tk expression cassette for negative selection was linearized with the restriction enzyme PacIand electroporated into C57BL/6 derived B6/BLU embryonic stem (ES) cells. 96 ES cell clones were selected and verified for correct recombination with long-range PCR and Southern blot analysis. We injected cells from a positive ES clone 3G into C57BL/6J blastocysts and then transferred them to pseudopregnant mice. Chimeric male mice displaying > 50% coat color chimerism were bred to C57BL/6 N females to generate F1 offspring. Germline transmission of the targeted *Car1* allele (defined as an Fln allele) was verified by PCR analysis of tail DNA from F1 offspring. Chimeric mice and WT C57BL/6J mice were mated to obtain heterozygous Fln/WT mice.

Next, we used heterozygous Fln/WT mice breeding with CMV-Cre mice to get *Car1*^*+/−*^/*Cre*^*+/−*^ line and backcross *Car1*^*+/−*^/*Cre*^*+/−*^ with WT to remove Cre and get *Car1*^*+/−*^. We allowed *Car1*^*+/−*^mice self-crossing to get littermate WT control, heterozygous and homozygous mice for Car1KO mice. We used two pairs of primers to confirm these three possibilities. 1) Car1-FRT-tF1 (5’- ATAGTGGCTTGCCCATTAAAACACC-3’) and common En2-R (5’- CCAACTGACCTTGGGCAAGAACAT-3’). A band of 342 bp indicates that this mouse carries at least one partial Fln allele; no band means a WT mouse. 2) Car1-loxP-tF1 (5’-TGAGCACACCGTGGATGGAACT-3’) and Car1-loxP-tR1 (5’- GATGTACGTGTGTTCAGTTTTTCAC-3’). A band of 516 bp indicates that this mouse has at least one WT allele; a band of 634 bp means that the mouse is carrying at least one Fln allele (the absence of this band is to ensure the knockout of *Car1*); no band means a homozygous mouse. After the removal of exon3, the product of the whole *Car1* gene will be eliminated which was confirmed by the result from the Western blot.

### Chronic social defeat stress (CSDS)

The CSDS procedure was performed similarly with our previous studies [36]. Briefly, CD1 mice undergoing screening for three days were used as resident aggressors. C57BL/6 mice were divided randomly into the CSDS group and control group. C57BL/6 mice in the CSDS group were defined as intruders in the experiment. After the habituation of two weeks for the resident CD1 mouse, the intruder mouse was introduced into that cage and exposed to direct physical contact with the resident CD1 mouse for 5 min. The intruder was then separated from the resident by a transparent perforated divider in the same cage until being transferred to another one for a new resident CD1 the next day. The whole process of social defeat for the CSDS group lasted for ten consecutive days.

### Chronic restraint stress (CRS)

Animals in the CRS group were independently placed into 50ml-plastic tubes with a few holes to keep airflow for 14 consecutive days (4-8 h per day, begin at 11:00 a.m. each day), while the control group received the same treatment for food and water deprivation without restraint.

### Chronic unpredictable mild stress (CUMS)

Male Sprague-Dawley rats were group-housed (four per cage) under a 12-h light-dark cycle with constant temperature (25 °C) and appropriate humidity. Rats have *ad libitum* access to food and water except for the fasting period). All rats were age (8 weeks) and weight-matched (250 ± 20 g) before CUMS procedures. Before any treatments or experiments, animals were allowed at least one week of habituation to the housing condition. Rats were then exposed to 9 different stressors in the next six weeks. Stressors are classified into long or short categories. The long ones include food and water deprivation, co-housed, inversed the circadian, soiled cage, tilting cage, and stroboscopic illumination, and the short ones include pinching tail, foot-shock, and shaking cage. A combination of a long stressor and a short one was used for CUMS every day. It was randomly composed each day and switched daily. For detailed descriptions, please refer to reference [5].

### Behavioral assay

Behavioral analyses were carried out by someone blinded to experimental conditions of mice during the light phase (13:00–18:00) in a soundproof room. All the animals’ behaviors were videotaped and measured by video tracking software (Ethovision, Noldus).

### Forced swim test (FST)

FST was used to evaluate the depression-like behavior by measuring time spent immobile. Mice were placed separately in a transparent glass cylinder (30 cm in height, 15 cm in diameter) filled with 20 cm of height tap water (23 ± 1 ℃). Tap water would be changed after each trial. Mice stayed in the cylinder for at least six minutes. The first two minutes were considered as an adaptation period. The duration of immobility in the next four minutes was measured automatically by a video-tracking system.

### Tail suspension test (TST)

The TST was conducted by hanging each mouse from the top of a white square box (20 cm×30 cm) for 6 min. The first two minutes were for adaptation. The duration of immobility was measured automatically for the next four minutes by a video-tracking system.

### Western blot assay

Mouse brain tissues in RIPA lysis buffer were ground in a grinding machine at 70 Hz for 120 s. Total protein extracts from mouse brain tissues or serum were first placed on ice for 20 min and then centrifuged at 12,000 rpm for 10 min at 4 ℃. Membrane proteins were further extracted with membrane and cytosol protein extraction kit (Cat: P0033, Beyotime) according to the user manual. The deactivated protein sample was separated in 10% SDS-PAGE and transferred into polyvinylidene fluoride membranes. After incubated with primary antibodies and then horseradish peroxidase-conjugated secondary antibodies, immunostained protein bands were detected with enhanced chemiluminescence in ChampChemi (Sagecreation). Primary antibodies used were rabbit anti-Car1 (1:2000, Abcam, Cat: ab108367), mouse anti-β-actin (1:10000, Proteintech, Cat: 66009-1-Ig), mouse anti-GAPDH (1:10000, Proteintech, Cat: 60004-1-Ig); secondary antibodies used in experiments were (goat Anti-Mouse IgG (H + L)-HRP Conjugate (1:10000, Bio-Rad, Cat:1,706,516), goat Anti-Rabbit IgG (H + L)-HRP Conjugate (1:8000, Bio-Rad, Cat:1,706,515). All bands were analyzed with Quantity One.

### Immunohistochemistry

Animals were anesthetized using 1% pentobarbital sodium and then perfused transcardially with ice-cold PBS (pH 7.4) followed by 4% paraformaldehyde (PFA). Mouse brains were fixed with 4% paraformaldehyde solution for 6 h and were dehydrated in 30% sucrose for one day at 4 °C. Coronal brain sections (20 μm) were cut with a microtome (Leica), bonded on glass slides for immunostaining in choroid plexus, and stored in 0.1 M PBS for immunostaining in the hippocampus. The sections were incubated with primary antibody at 4 °C overnight. After washing with PBS three times, sections were incubated with the secondary antibody. The antibodies used were goat anti-TTR (1:50, LifeSpan, Cat: 395,788), rabbit anti-CAR1 (1:200, Proteintech, Cat:13198-2-AP), mouse anti-NeuN (1:500, Abcam, Cat: ab104224), goat anti-GFAP (1:1000, Abcam, Cat: ab53554), mouse anti-S100β (1:1000, Sigma, Cat:AMAB91038), goat anti-Iba1 (1:100, Abcam, Cat: ab5076), mouse anti-MBP (1:500, Abcam, Cat: ab254026), Alexa Fluor 488 donkey anti-rabbit IgG (Cat: R37118), Alexa Fluor 555 donkey anti-goat IgG (Cat: A-21,432), Alexa Fluor 594 donkey anti-mouse IgG (Cat: A-11,058) (all 1:1000 and all is from Invitrogen). All fluorescent images of the mounted sections were captured with a Nikon A1R confocal microscope.

### RNAscope

Mice were anesthetized and then perfused with DEPC-treated PBS and 4% PFA. Brains were swiftly removed and post-fixed in 4% PFA for 1 day before dehydration in 30% sucrose. Frozen brain slices with 20 μm thickness at a similar coronal position were subjected for the RNAscope assay. RNAscope Multiplex Fluorescent Reagent Kit v2 (Advanced Cell Diagnostics, USA) was used for identifying *Car1* distribution according to the manufacturer’s standard protocol. We further performed immunostaining with an antibody against GFAP (1:500, Abcam, Cat: ab53554) after RNAscope assay to confirm the expression of *Car1* in astrocytes.

### Stereotactic surgery

Before virus injection, mice were fixed in a stereotaxic frame (RWD) after being anesthetized with isoflurane. We injected 1 µl of AAV virus into mouse ventral hippocampus (vHPC) (AP: -3.3 mm from bregma, ML: ± 3.0 mm, DV: -1.5 mm/-2.5 mm/-3.5 mm from the brain surface) bilaterally with glass microelectrodes. To label the choroid plexus, 4 µl of AAV5 virus was injected slowly into the right lateral ventricle (AP: -0.4 mm from bregma, ML: -1.0 mm, DV: -2.0 mm from the brain surface). After injection, the glass microelectrode was maintained in the injected place for an additional 3 min and then was withdrawn gently afterward. We sutured the wound transferred mice back to their home cage. Behavior tests or electrophysiology recordings were not conducted in the first 3-weeks after the surgery.

### Cannula infusion experiment

For delivering drugs, we inserted the guide cannula (RWD) into the right lateral ventricl**e (**AP: -0.4 mm from bregma, ML: -1.0 mm, DV: -2 mm from the brain surface) or bilaterally ventral DG (AP: -3.28 mm from bregma, ML: ± 3.75 mm, DV: -1.9 mm from the brain surface) at a 15° angle to the coronal plane of mice. At least one week after mice had recovered from the surgery, L-phenylalanine (25 µg/µl, dissolved in 0.9% saline) was microinjected with a cannula gauge injector, which has a 0.1-mm extension beyond the tip of the guide cannula. The same volume of saline was used for the control mice. After all behavioral tests, we verified injection sites with another injection of 1 µl CTB-488 (Invitrogen, US).

### L-Phe administration

L-Phe (Sigma, Cat: P2126) was freshly prepared and dissolved in0.9% saline. The drug was administrated daily through intraperitoneal injection (i.p) with the dosage of 10 mg/kg and 15 mg/kg at 15:00 daily for 35 consecutive days. Antidepressant response to systemic administration of L-Phe (25 µg/side/day) via dual-guide cannulae into lateral ventricle at fixed timing during a day (13:00) for ten consecutive days then performed forced swimming test.

### pH ACSF administration

Aldult male C57B/J mice and CAR1^−/−^ mice were anaesthetized with 0.8–1.5% isoflurane from a vaporizer and fixed on a stereotaxic apparatus. Guide cannulas made of stainless steel tubing were implanted into the bilateral ventral hippocampus (-3.28 mm AP; ±3.75 mm ML; -1.95 mm DV from bregam). Before the behavioural experiments, 7 days was allowed for mice to recovery. Intra-hippocampal injection was performed via insertion of needles by the cannulas. Different pH ACSF solutions were administered in a total volume of 2 µl each side. To allow drug diffusion, the needles were left in place for an additional 3 min after injection.

### Measurement of CAR activity

Male adult C57B/J6 mice were randomly divided into two groups and treated with saline (i.p.) or L-Phe (15 mg/kg) (n = 5). 60 min after drug administration into the brain, mice were quickly sacrificed, and their brains were dissociated on ice, then immediately homogenized in 1mL of ice-cold 20mM HEPES buffer (pH7.5). After the centrifugation at 20,000 g for 30 min, we used the photophysics stopped-flow spectrometer for evaluating the CA-catalyzed CO_2_ hydration activity (Shanghai Yuduo biotech, Cat: GMS508462).

### The indication of pH changes in the DG by the pH-sensitive dye pHLIP

To measure the pH changes in the DG induced by the absence of CAR1, herein we synthesized a peptide, which is a pH (low) Insertion Peptide (pHLIP: AAEQNPIYWARYADWLFTTPLLLLDLALLVDADEGTCG), to conjugate rhodamine B at its C terminus by a disulfide bond. Once sensing the acidic extracellular environment, this peptide could permeate a membrane and form a transmembrane α-helix. Furthermore, the impaired disulfide would release the rhodamine B into the cytoplasm of living cells. Thus, the pHLIP peptide is selective for indicating low pH extracellular medium [2, 26]. The pHLIP peptide was synthesized (by BoTech, Shanghai, China) in a typical preparation of the water-soluble form of the peptide. The lyophilized powder was dissolved in a solution containing 6 M urea, 200mM NaCl, 10mM Tris, pH 8.3 at approximately 0.5 mg/mL. The peptide solution was dialyzed twice against 300 volumes of the same buffer without urea and then dialyzed three times against 300 volumes of 20mM NaCl, 5mM NaPO_4_, pH 8.0, then yielding the final working concentration approximate to 0.5 mg/ml.

For pHLIP injection, the stereotactic surgery procedures and injected parameters are the same as to aforementioned viral injection. 2 µl pHLIP (7 μm) was injected into the ventral hippocampus (AP: -3.3 mm from bregma, ML: ±3.0 mm, DV: -1.5 mm/-2.5 mm/-3.5 mm from the brain surface) with glass microelectrodes. 24 h after injection, mice were deeply anesthetized and perfused with phosphate-buffered saline (PBS) followed by 4% paraformaldehyde. Brains were post-fixed with 4% PFA overnight plus cryoprotected by immersion in 30% sucrose solution (in PBS). Frozen mice brains were sectioned with a thickness of 60 μm. Fluorescent images were acquired by VS120 (Olympus) and quantified with Image J software. The fluorescence density of pHLIP in the DG hilus cells from the same slice will be averaged and counted as one data set in the quantification.

### Acute hippocampal slice preparation

In all cases, animals were anesthetized using pentobarbital sodium (90 mg/kg) administered intraperitoneally. Intracardiac perfusion was then performed using NMDG-based artificial cerebrospinal fluid (NMDG-aCSF) solutions, which containing (in mM): NMDG 93, KCl 2.5, NaH2PO4 1.2, NaHCO3 30, MgSO4 10, CaCl2 0.5, HEPES 20, D-glucose 25, Na-ascorbate 5, thiourea 2, Na-pyruvate 3 and N-acetyl-L-cysteine 12 (pH was adjusted to 7.4 using HCl). Then 300 μm-thick horizontal slices were obtained using a Leica VT1200S vibratome and transferred swiftly to the 32 °C preheating NMDG aCSF for at least 12 min. Afterward, slices were transferred to a holding chamber which contained HEPES-aCSF (in mM): NaCl 93, KCl 2.5, NaH2PO4 1.2, NaHCO3 30, MgSO4 10, CaCl2 0.5, HEPES 20, D-glucose 25, Na-ascorbate 5, thiourea 2, Na-pyruvate 3 and N-acetyl-L-cysteine 12 (pH was adjusted to 7.4 using NaOH). Slices were incubated for an hour minimum at room temperature before recordings. Normal aCSF composed of (mM): NaCl 126, KCl 3, NaH2PO4 1.2, NaHCO3 26, MgCl2 1.3, CaCl2 2.6, D-glucose 10 was used as a recording solution unless indicated otherwise. All aCSFs (300 and 310 mOsm) were bubbled with 95% O2/5% CO2 gas. Slices were constantly perfused at a rate of 2 ml/min with normal aCSF.

### Electrophysiology

A differential interference contrast microscope (BX51W1, Olympus), a charge-coupled device camera, and a 5X/NA 0.1 objective and a 40X/NA 0.8 W objective were used to visualize cells and electrodes for whole-cell patch-clamp recording. Recordings were obtained with an amplifier (MultiClamp 700B, Molecular Devices) coupled to a digitizer (Digidata 1440 A, Molecular Devices). Borosilicate glass pipettes with inner filament (OD 1.5 mm, ID 0.86 mm; Sutter Instrument) were pulled by a micropipette puller (P-97; Sutter Instrument), and the ones with resistance at 3.5–7MΩ were used for recordings. The temperature of the recording fluid was controlled at 30–32℃ by the temperature controller (TC-344 C, Warner Instruments). Slices were transferred from the holding solution to the recording chamber with normal aCSF, and recording pipettes were filled with an intracellular solution containing (in mM): CsCl2 140, HEPES 10, MgCl2.6H2O 4.25, CaCl2 0.15, EGTA 0.5, Na2-ATP 4, QX-314 2 (pH 7.3 and osmolality 290–295 mOsm). We clamped granule cells holding at − 60mV. Cells with a series resistance less than 30MΩ and a leaking current less than − 50pA were kept for analysis. To record the miniature excitatory postsynaptic current (mEPSC), we added TTX (1µM) and picrotoxin (100µM) into the recording chamber. For recording miniature inhibitory postsynaptic current(mIPSC), we applied TTX (1µM), NBQX (10µM), and AP-5 (100µM) in the chamber to block the potential effects of action potentials and sEPSCs. To record the granule cells’ responses upon the stimulation of the perforant pathway, we placed a bipolar stimulating electrode at the DG molecular layer. Pulse stimulations with 0.1ms width were given at 40HZ during 500ms by using a stimulus isolator (ISO-flex, AMPI instrument). When aCSF without HCO3^−^ was adopted, we used an equal molar mass of NaCl to replace NaHCO3 and bubbled pure O2(> 99.99%) in the HEPES buffer solution. Recordings were filtered at 2 kHz, digitized at 20 kHz, and stored with the pClamp 10 software suite (Molecular Devices). Patch-clamp data were analyzed with Clampfit 10.9 (Molecular Devices) and Mini Analysis 6 software (Synaptosoft) in a semi-automated fashion (automatic detection of events with chosen parameters followed by a visual validation).

### NMDAR/AMPAR ratio measurement

First, we placed the stimulating electrode at the EC region and recorded currents of GCs. The stimulating intensity was optimized through the I/O curve, and the one to get 70-80% of the maximum current was used. We used the picrotoxin (100µM) to isolate EPSCs, and recorded AMPAR mediated EPSCs at -70mV. CNQX (20µM) was further added in the recording chamber to block AMPAR to get total NMDAR-mediated EPSCs at + 40mV. Afterward, we continue adding RO25-6981(0.5µM) to separate NR2A-contained NMDAR-mediated EPSCs.

### LTP/LTD induction

We placed the stimulating electrode at the CA3 of vHPC and recorded the field excitatory postsynaptic potential (fEPSP) in the CA1 region through a glass electrode filled with a solution of 3 M NaCl. The stimulus intensity was optimized through the I/O curve, and the one to get 50% of the maximum current was used. At the beginning of the experiment, fEPSP was induced every 1 min and recorded stably for at least 30 min, then the TBS or LFS protocol was adopted to induce LTP or LTD, respectively. After that, the fEPSP was recorded for at least 60 min.

### Statistical analysis

All results were presented as the means ± s.e.m. GraphPad Prism software V7 One-way ANOVA or independent-sample *t-test* were used for statistical analyses in this study.

## Electronic supplementary material

Below is the link to the electronic supplementary material.

Supplementary Material 1

Supplementary Material 2
