# Supplementary material for: Upregulation of carbonic anhydrase 1 beneficial for depressive disorder
Source: Acta Neuropathol Commun. 2023 Apr 3;11:59. doi: 10.1186/s40478-023-01545-6 (PMC10071615; doi:10.1186/s40478-023-01545-6)
Supplement: Supplementary file 2 — Supplementary Material 2 [file 40478_2023_1545_MOESM2_ESM.docx]

**Supplemental Table 1. The viruses used in the study.**

| **Viral vector** | **Type** | **Titer (TU/ml; VG/ml)** | | **Dilution** |
| --- | --- | --- | --- | --- |
| pLenti-Ubc-mCherry-CAR1-2A-MCS-3FLAG | Lentivirus | | 9.64E+08 | / |
| pLenti-Ubc-mCherry-2A-MCS-3FLAG | Lentivirus | | 1.19E+09 | / |
| pAAV-shortGFAP-EGFP-P2A-CAR1-3FLAG | AAV2/8 | | 4.27E+12 | / |
| pAAV-shortGFAP-MCS-EGFP-3FLAG | AAV2/8 | | 2.18E+13 | 1:5 |
| pAAV-SYN-EGFP-P2A-CAR1-FLAG | AAV2/8 | | 5.40E+12 | / |
| pAAV-SYN-EGFP-P2A-MCS | AAV2/8 | | 5.14E+12 | / |
| pAAV-CMV-eGFP-U6-shRNA (CAR1) | AAV2/5 | | 6.17E+12 | / |
| pAAV-CMV-eGFP-U6-shRNA (NC3) | AAV2/5 | | 1.58E+13 | / |

**Supplemental Table 2. Clinical characteristics of the MDD patients and controls**

| **Characteristics** | **Drug-naive MDD** | **Healthy controls** | **P-value** |
| --- | --- | --- | --- |
| Patients (n) | 35 | 36 | / |
| Male (n, %) | 17 (48.6%) | 17 (47.2%) | 0.722 |
| Age (Mean ± S.E.M.) | 26± 0.50 | 25.63± 0.42 | 0.578 |
| HAMD-17 (Mean ± S.E.M.) | 21.7 ± 0.64 | NA | / |


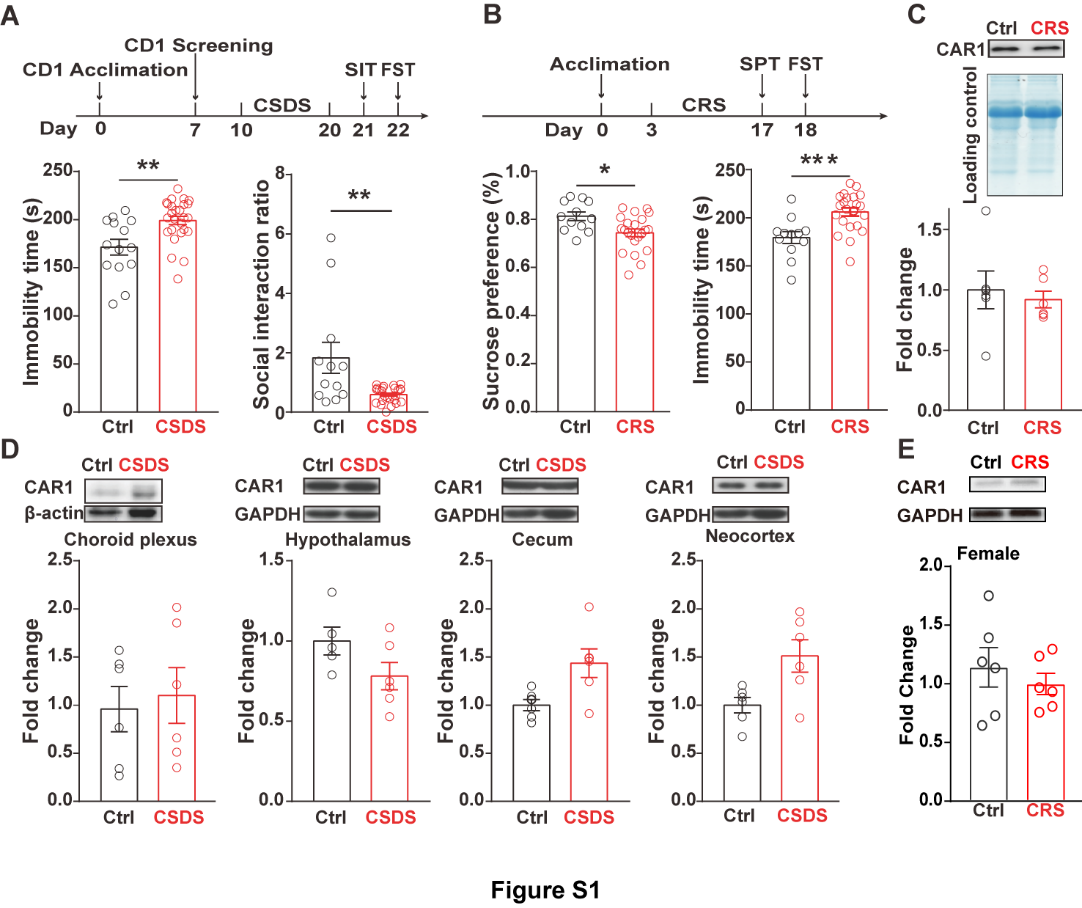


**Supplemental Figure 1**: **Changes of CAR1 in different brain areas of depression-like model rodents. (A)** Schematic of CSDS paradigm for mice (top). The immobility time in FST and the social interaction time (SIT) for control (n=14 for FST and n=12 for SIT) and CSDS treated mice (n=26) (bottom). **(B)** Schematic of CRS paradigm for mice (top). The sucrose preference and the immobility time in FST for control (n=12) and CRS-treated mice (n=22) (bottom). **(C)** Western blot validation of CAR1 in the serum of CRS mice model comparing to control group, total protein used as a loading control. **(D)** Western blot analysis showed the changes in the choroid plexus, hypothalamus, cecum, and neocortex in the CSDS-treated mice compared to the control group (n=6). **(E)** Western blot validation of CAR1 in the hippocampus of female CRS mice model comparing to female control group. Asterisk indicate the statistically significant differences. ** P<0.01, *** P<0.001 compared to control groups.


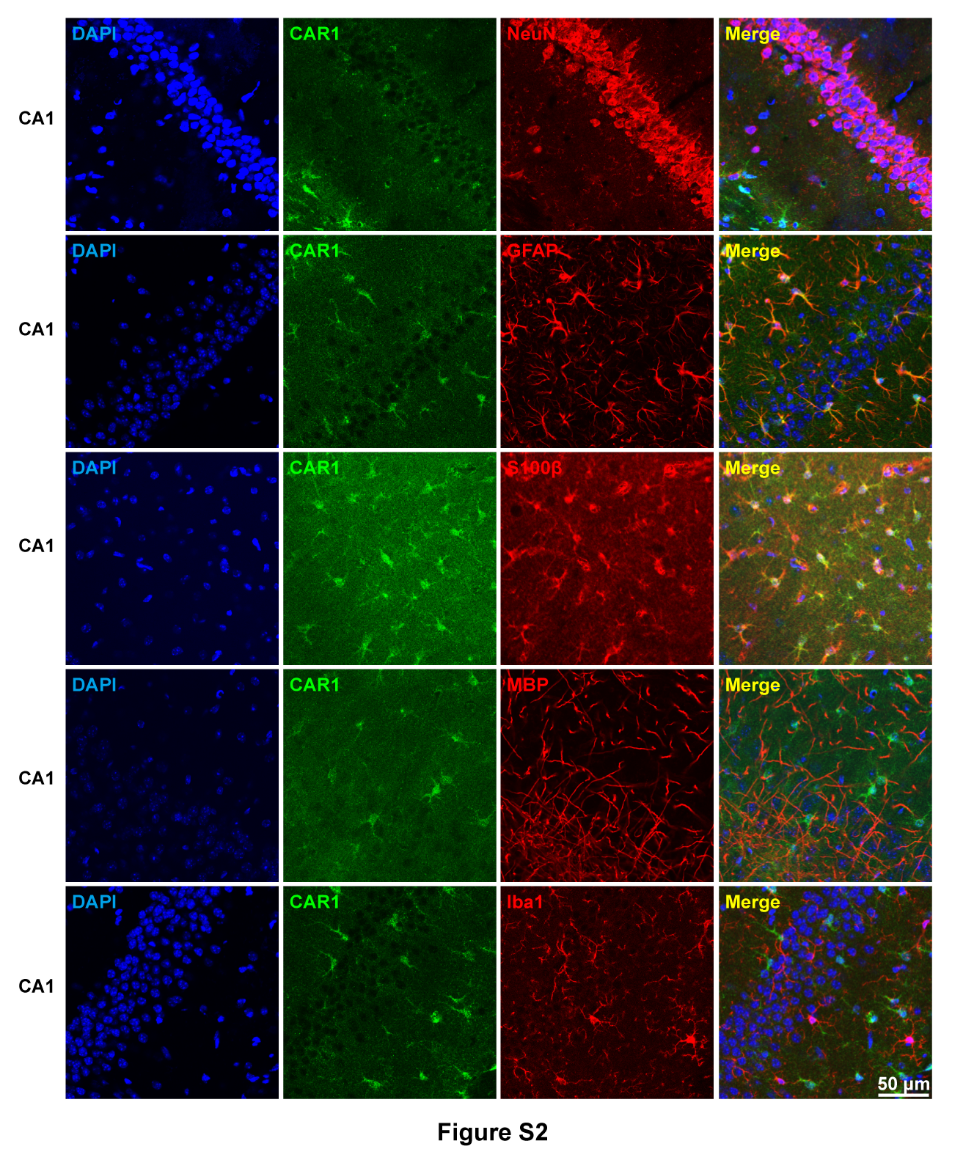


**Supplemental Figure 2**: **CAR1 Expression in astrocytes of hippocampal CA1.** Co-immunostainings with antibodies against CAR1 (green) and different cell type markers (red) such as neuronal nuclei antigen (NeuN, neuron), glial fibrillary acidic protein (GFAP, astrocytes), S-100 protein subunit beta (S100β, astrocytes), myelin basic protein (MBP, oligodendrocytes) and ionized calcium-binding adapter molecule 1 (Iba1, microglia). Scale bars, 50μm.


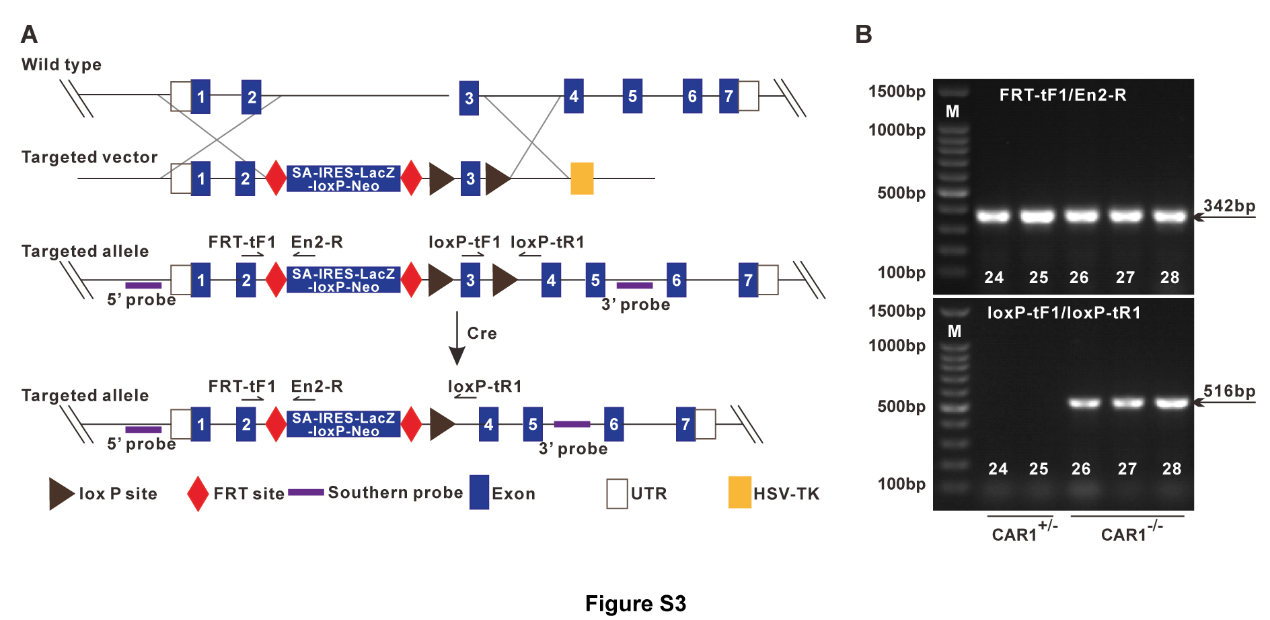


**Supplemental Figure 3**: **Generation of *CAR1* knockout mice. (A)** Targeting strategy for knocking out exon III of the *Car1*. **(B)** Genotyping PCR of *CAR1*^-/-^ mice. Mice 24 and 25 were heterozygous and mice 26, 27, and 28 were homozygous for *CAR1*^-/-^ mice.

**
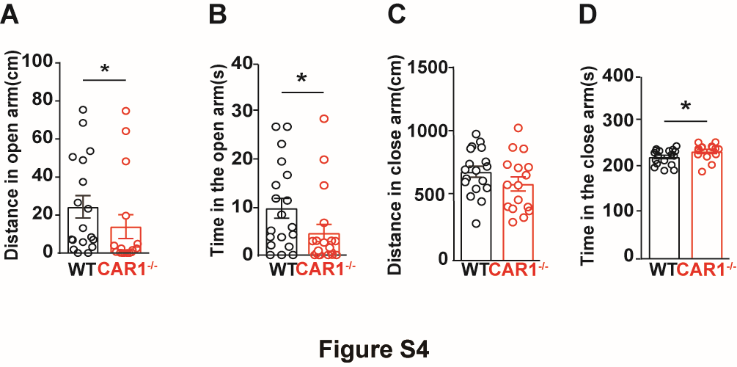
**

**Supplemental Figure 4**: **Elevated plus maze of CAR1 and WT mice. (A)** Distances in the open armed of EPM between CAR1^-/-^ mice and WT mice; **(B)** time spent in the open armed of EPM between CAR1^-/-^ mice and WT mice; **(C)** Distances at closed armed in the EPM between CAR1^-/-^ mice and WT mice; **(D)** time spent in the open armed of EPM between CAR1^-/-^ mice and WT mice;

**
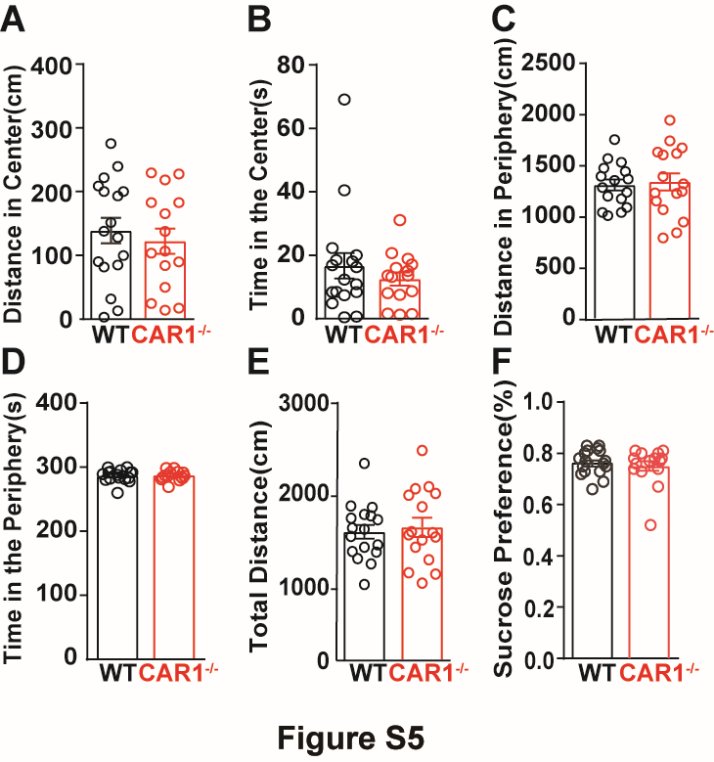
**

**Supplemental Figure 5：Open field test and sucrose preference test for CAR1**^-/-^ and WT **mice. (A)** Distances in the open armed of EPM between CAR1^-/-^ mice and WT mice; **(B)** time spent in the open armed of EPM between CAR1^-/-^ mice and WT mice; **(C)** Distances at closed armed in the EPM between CAR1^-/-^ mice and WT mice; **(D)** time spent in the open armed of EPM between CAR1^-/-^ mice and WT mice;

**
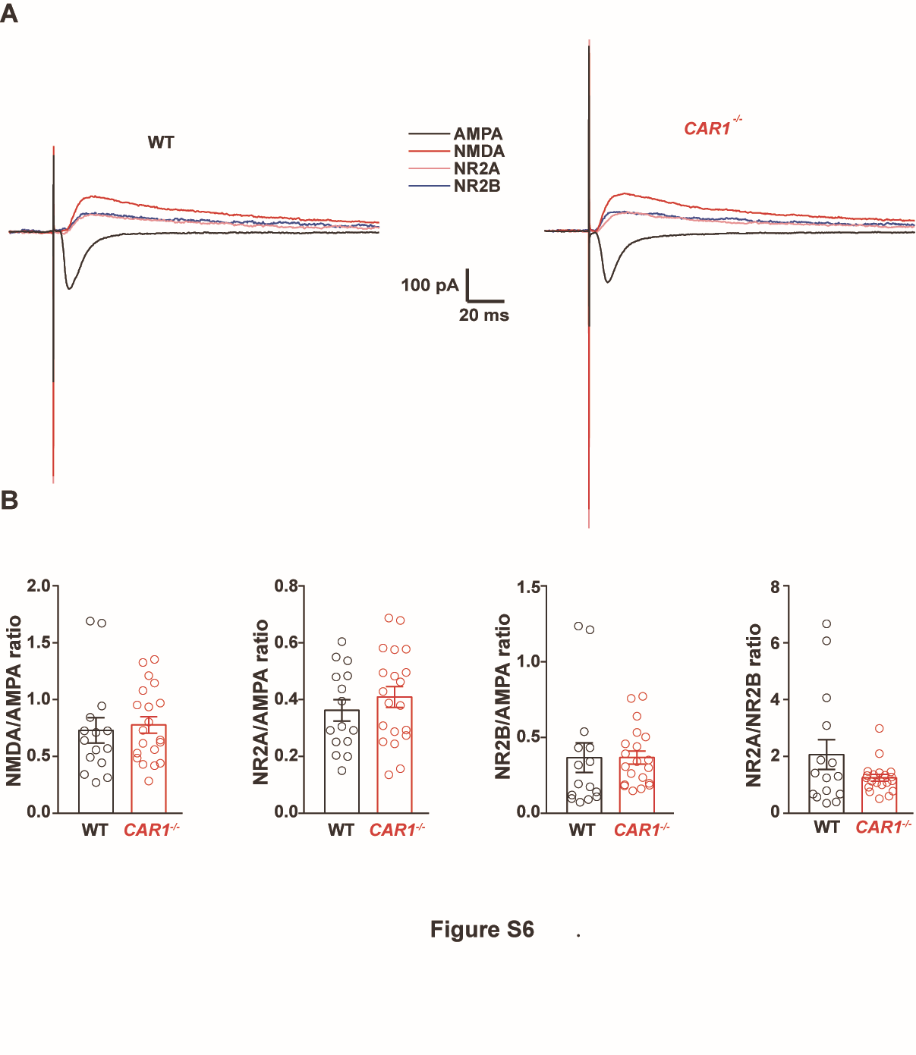
**

**Supplemental Figure 6**: **Evoked EPSC in WT mice and *CAR1^-/-^* mice. (A)** Representative traces of evoked EPSC at +40mV (total NMDA red, NR2A-NMDA pink, and NR2B-NMDA blue) and −70mV (AMPA, black) in the presence of picrotoxin (100μM) are shown for granule cells from WT and CAR1^-/-^ mice, respectively. **(B)** Histograms with dot plots summarizing the current amplitude ratio of AMPA/NMDA, NR2A /AMPA, NR2B /AMPA**,** and NR2A/NR2B of granule cells from WT and *CAR1^-/-^* mice.


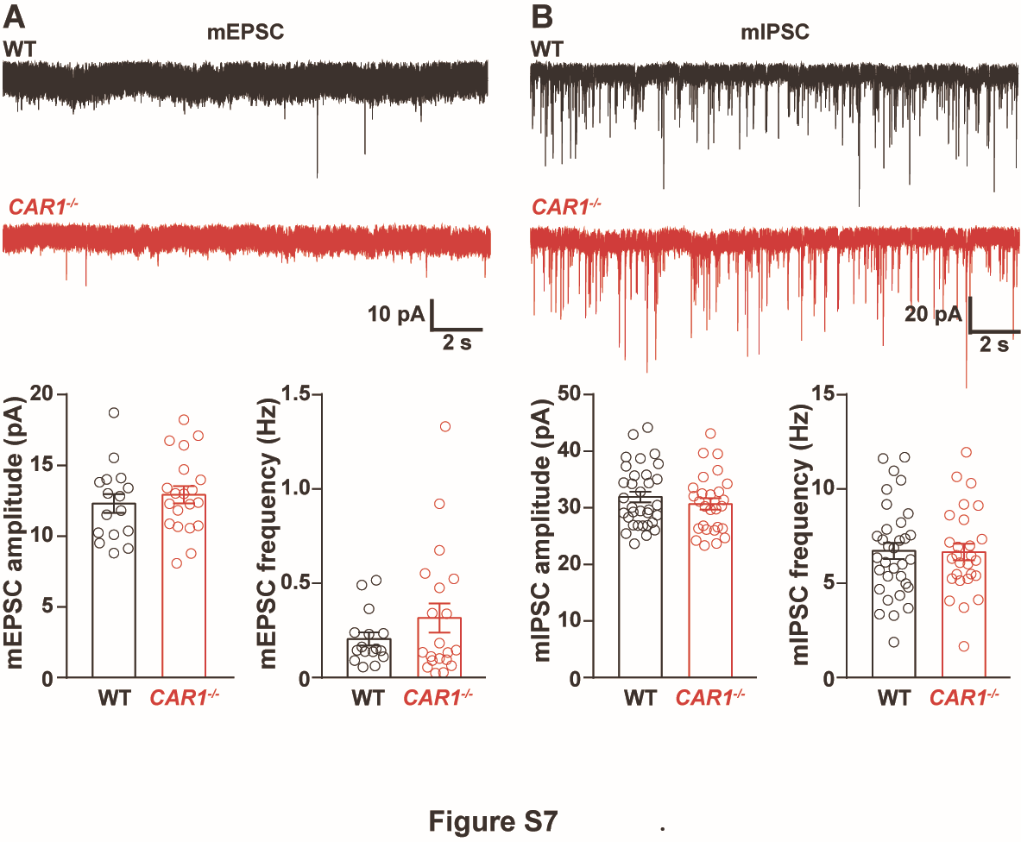


**Supplemental Figure 7**: **Evaluation of E/I in CA1 pyramidal neurons. (A)** Representative traces of mEPSCs of CA1 cells from WT and *CAR1*^-/-^ mice (top**)**; average mEPSCs amplitude and frequency of CA1 pyramidal cells in WT mice and *CAR1*^-/-^ mice (bottom). **(B)** Representative traces of mIPSCs of CA1 cells from WT and *CAR1^-/-^* mice (top**)**; average mIPSC amplitude and frequency of CA1 pyramidal cells in WT mice and *CAR1*^-/-^ mice (bottom).

**
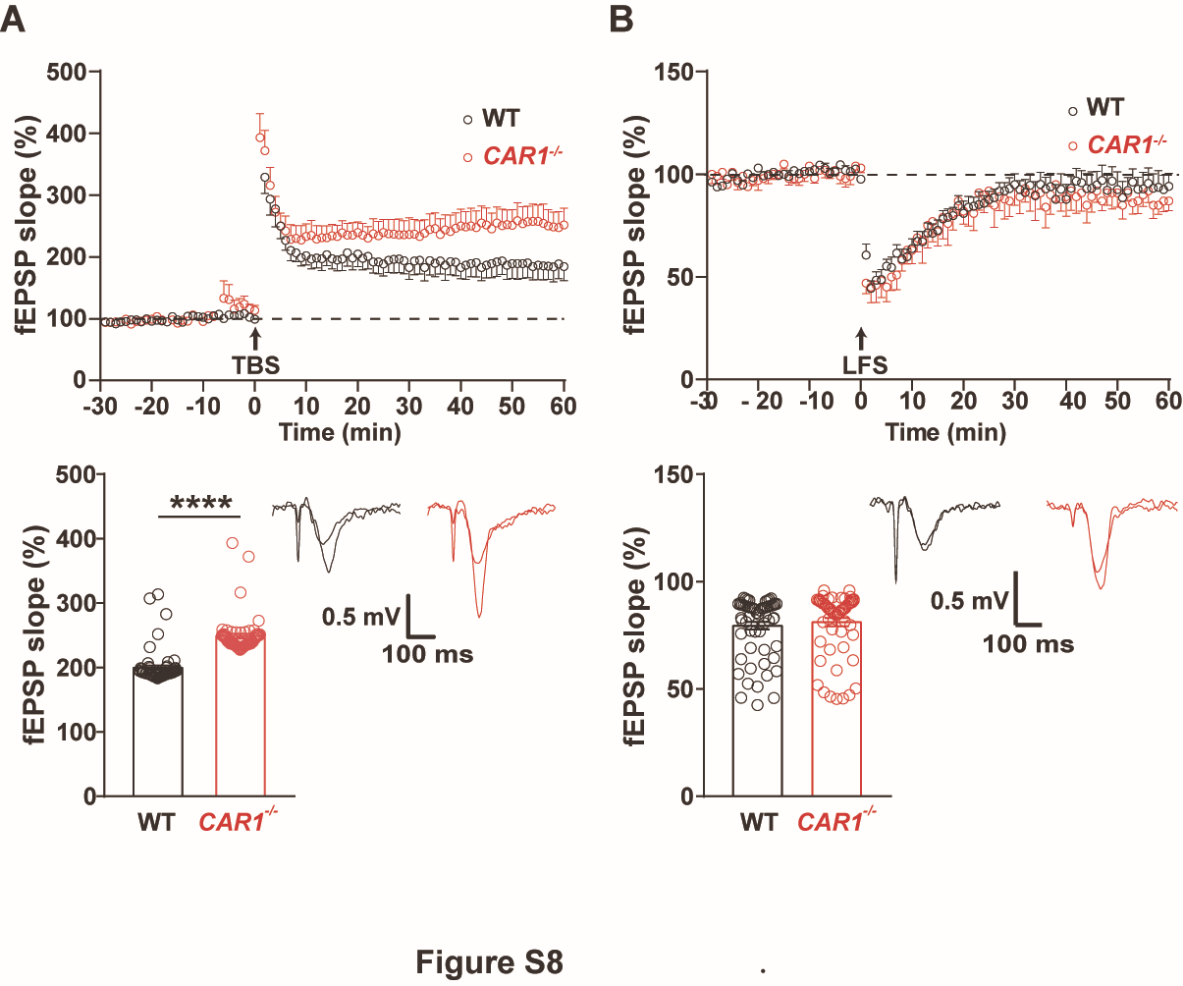
**

**Supplemental Figure 8**: **Hippocampal LTP and LTD recorded from brain slices of WT and *CAR1*^-/-^ mice. (A)** Hippocampal LTP is enhanced in *CAR1*^-/-^ mice compared to WT. The plot (top) presents normalized slopes of fEPSPs; the bar graph (bottom) summarizes the average percentage of fEPSP change at 5 minutes before and 55 minutes after the TBS delivery, and corresponding representative traces showing at the right. **(B)** Hippocampal LTD remains unchanged in *CAR1*^-/-^ mice compared to WT. The plot (top) presents normalized slopes of fEPSPs; the bar graph (bottom) summarizes the average percentage of fEPSP change at 5 minutes before and 55 minutes after the LFS delivery, and corresponding representative traces showing at the right.

**
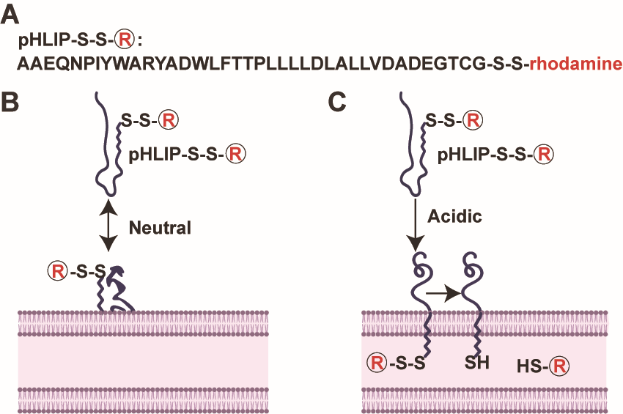
**

**Supplemental Figure 9**: **Schematic diagram showing how pHLIP–rhodamine transport into a cell.** Under the physiological pH condition, the peptide pHLIP–rhodamine barely interacts with a membrane (left). Under the acidic pH condition, the peptide forms a transmembrane helix with its C terminus and inserts into the cytoplasm, and then red rhodamine will be detached from the peptide at the breakage of the disulfide bond (right).


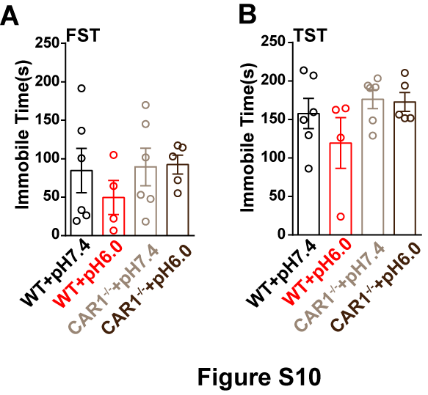


**Supplemental Figure 10: Forced swimming test and tail suspension test after Ph6.0/Ph7.4 ACSF administration at vDG. (A)** immobility time in FST between WT and CAR1^-/-^ mice. **(B)** immobility time in TST between WT and CAR1^-/-^ mice.


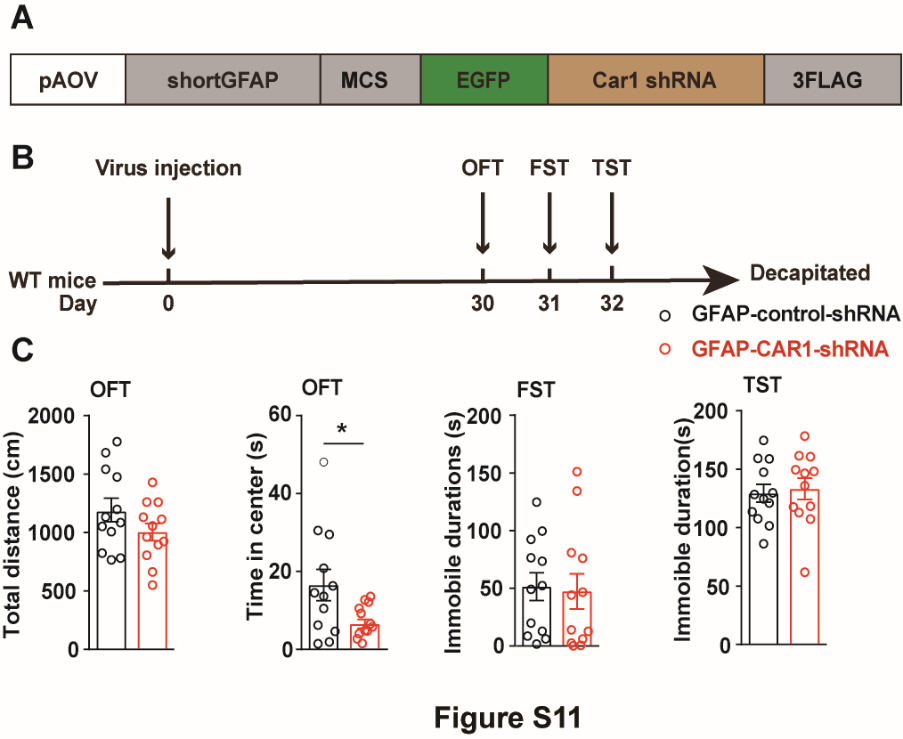


**Supplemental Figure 11: GFAP-driven interference of car1 in the vHPC of WT mice. (A)** Depiction of AAV engineered to locally car1-shRNA that expresses EGFP. **(B)** Experimental paradigm for stereoscopic injection and behavioral tests. **(C)** No significant differences were found between the GFAP-car shRNA and GFAP-ctrl groups. Data are expressed as mean ± s.e.m. (n=9-10 mice per group).


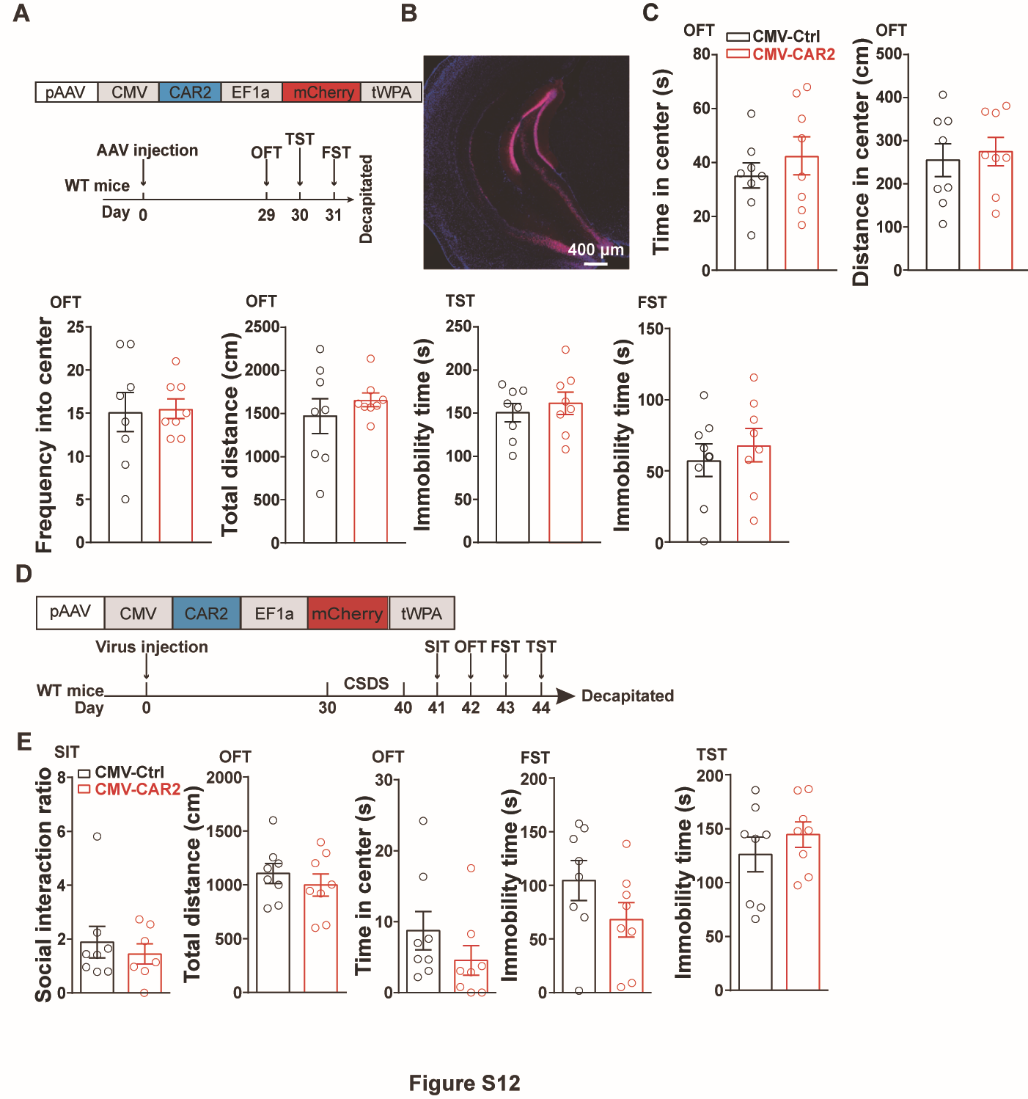


**Supplemental Figure 12**: **Behavior evaluation after overexpressing CAR2 in the vHPC with or without the CSDS treatment**. **(A)** Schematic representation of AAV construct showing mouse CAR2 under the transcriptional regulation of CMV promoter (top); the timeline of experimental procedure (bottom). **(B)** A representative image of the AAV injection site in the vHPC. **(C)** Behavior tests after AAV-Ctrl or AAV-CAR2 infection including OFT, FST, and TST (n=8 for both Ctrl and CAR1). **(D)** The timeline of CSDS experimental procedure after the injection of the same AAV virus as in (A). **(E)** Behavior tests after AAV-CAR2 infection coupling with CSDS treatment including SIT, OFT, FST, and TST (n=8 for Ctrl and n=7-8 for CAR1).


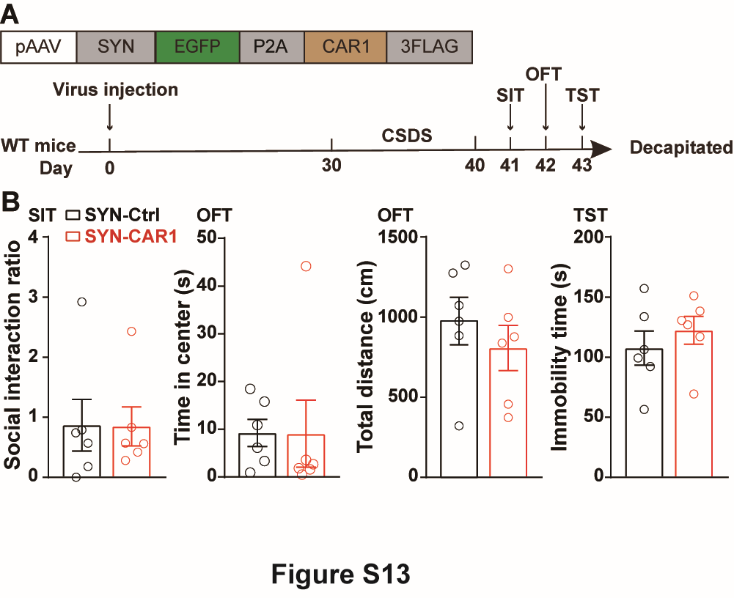


**Supplemental Figure 13**: Overexpression of CAR1 in the neurons of vHPC failed to prevent CSDS-induced depression-like behavior. **(A)** Schematics of an AAV vector expressing CAR1 under the SYN promoter (top), the experimental paradigm for behavioral testing **(**bottom)**.** **(B)** Behavior tests after AAV-CAR1 infection coupling with CSDS treatment including SIT, OFT, and TST. Data are expressed as mean ± s.e.m. (n=6).


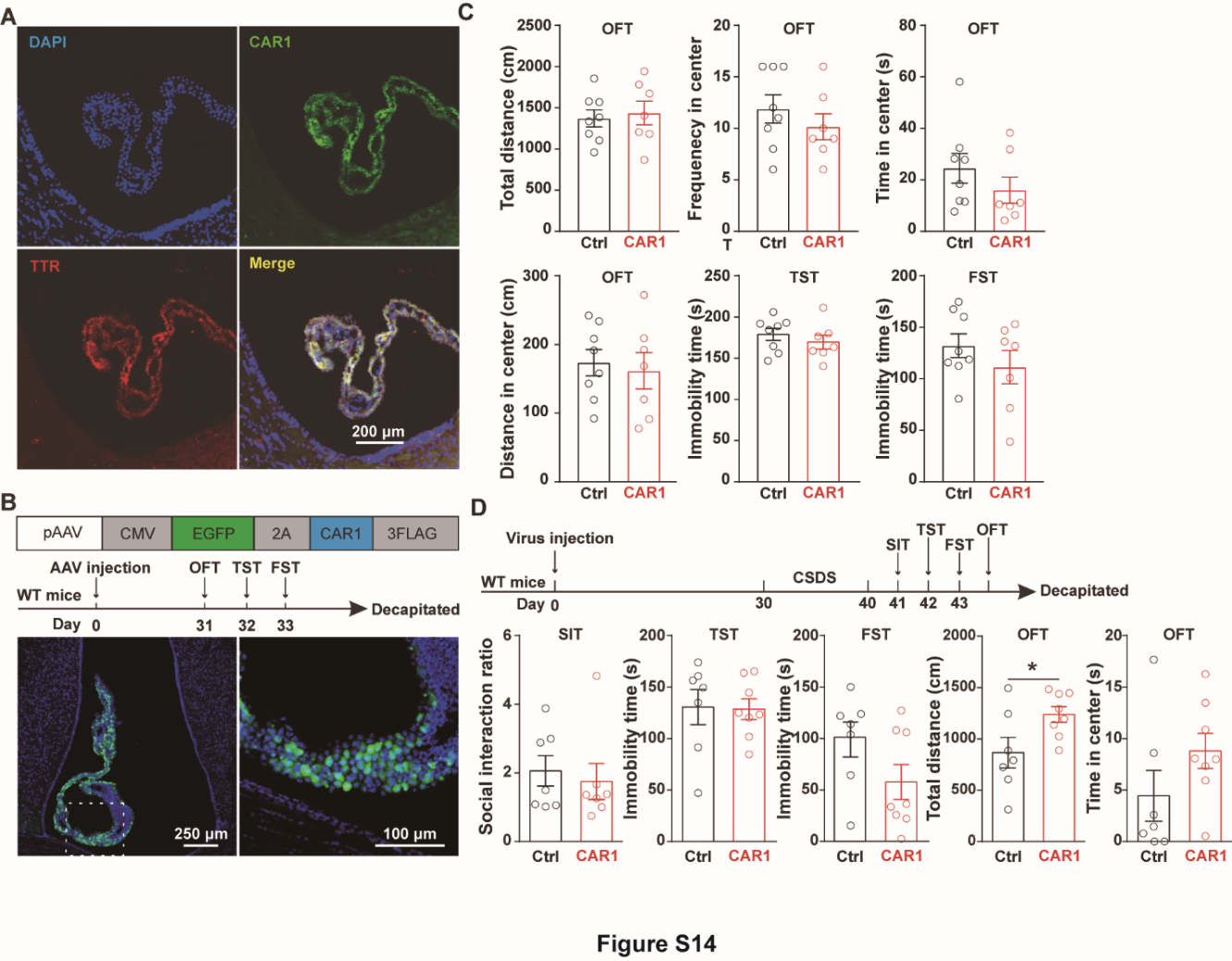


**Supplemental Figure 14**: **Overexpression of CAR1 in the choroid plexus did not affect depression-like behaviors. (A)** CAR1 expression in the choroid plexus. Co-immunostained transthyretin TTR (red) with CAR1 (green) to identify CAR 1 expression in CP epithelial cells and endothelial cells. Scale bars, 200μm. **(B)** Schematic representation of an AAV construct showing mouse CAR1 under the transcriptional regulation of CMV promoter (top), the timeline of experimental procedure (middle), representative confocal images of injection sites in the lateral ventricle after AAV5 infection (bottom). **(C)** Behavior tests after AAV5- CAR1 infection including OFT, TST, and FST (n=8 for Ctrl and n=7 for CAR1). **(D)** The timeline of experimental procedure (top). Behavior tests after AAV5-CAR1 injection coupling with CSDS treatment including SIT, OFT, FST, and TST (n=7 for Ctrl and n=7-8 for CAR1) (bottom).
